# The 100 most cited articles on malocclusion’s biopsychosocial impact in school-aged youth: bibliometric study

**DOI:** 10.1590/2177-6709.31.3.e2625242.oar

**Published:** 2026-08-28

**Authors:** Millena Barroso OLIVEIRA, Andrea Sayuri Silveira Dias TERADA, Danilo Cassiano FERRAZ, Marcelo Bighetti TONIOLLO, Walbert de Andrade VIEIRA, Saul Martins PAIVA, Rafael Rodrigues LIMA, Luiz Renato PARANHOS

**Affiliations:** 1 Universidade Federal de Uberlândia, School of Dentistry (Uberlândia/MG, Brazil).; 2 Universidade de Rio Verde, School of Dentistry (Rio Verde/GO, Brazil).; 3 University of Pittsburgh, School of Dental Medicine, Department of Endodontics (Pittsburgh/PA, USA).; 4 Centro Universitário das Faculdades Associadas de Ensino, School of Dentistry (São João da Boa Vista/SP, Brazil).; 5 Universidade Federal de Minas Gerais, School of Dentistry, Department of Pediatric Dentistry (Belo Horizonte/MG, Brazil).; 6 Universidade Federal do Pará, Institute of Biological Sciences, Laboratory of Structural and Functional Biology (Belém/PA, Brazil).; 7 Universidade Federal de Uberlândia, School of Dentistry, Department of Orthodontics (Uberlândia/MG, Brazil).

**Keywords:** Adolescent, Bibliometrics, Child, Malocclusion, Models, biopsychosocial, Adolescente, Bibliometria, Criança, Má oclusão, Modelos biopsicossociais

## Abstract

**Objective:**

This study assessed the characteristics of the 100 most-cited papers on the biopsychosocial impact of malocclusion in school-aged youth.

**Methods:**

The Web of Science Core Collection (WoS-CC) database was searched. Data extracted included title of the paper, year of publication, authorship, number of citations, institution, country, continent, study design, journal and its impact factor, and keywords. A content analysis was performed to extract information on malocclusion diagnosis indices, age group, sample size, biopsychosocial outcomes, and assessment instruments. Bibliometric networks were created using VOSviewer and Power BI software. Descriptive analyses and Poisson regression were conducted (*p* < 0.05).

**Results:**

The 100 most-cited articles were selected from the 1,297 studies, totaling 3,621 citations. Most of the studies were cross-sectional (n=89), with a majority originating from Brazil (n=54). *The Angle Orthodontist* and the *American Journal of Orthodontics and Dentofacial Orthopedics* stood out with the highest number of publications and citations (11 articles, 532 and 461 citations, respectively). The authors with the most citations was C.A. Feldens, being the most cited author (n = 256). “Malocclusion” was the most used keyword (n = 39). The most prolific institutions were *Universidade Federal de Minas Gerais* (12 articles), *Universidade Estadual de Campinas* (11 articles), and *Universidade Federal de Santa Maria* (8 articles).

**Conclusion:**

Cross-sectional studies conducted by Brazilian authors examining the impact of malocclusion on oral health-related quality of life predominated. More diverse study designs, broader geographic representation, and longitudinal approaches are necessary to strengthen the evidence on the biopsychosocial effects of malocclusion.

## RESUMO

**Introdução:**

Este estudo avaliou as características dos 100 artigos mais citados sobre o impacto biopsicossocial da má oclusão em crianças e adolescentes em idade escolar.

**Métodos:**

A base de dados Web of Science Core Collection (WoS-CC) foi pesquisada. Os dados extraídos incluíram título do artigo, ano de publicação, autoria, número de citações, instituição, país, continente, delineamento do estudo, periódico e seu fator de impacto, além de palavras-chave. Foi realizada análise de conteúdo para extrair informações sobre índices diagnósticos de má oclusão, faixa etária, tamanho amostral, desfechos biopsicossociais e instrumentos de avaliação. Redes bibliométricas foram criadas utilizando os *softwares* VOSviewer e Power BI. Foram realizadas análises descritivas e regressão de Poisson (*p* < 0,05).

**Resultados:**

Os 100 artigos mais citados foram selecionados entre 1.297 estudos, totalizando 3.621 citações. A maioria dos estudos foi transversal (n = 89), com predominância do Brasil (n = 54). *The Angle Orthodontist* e o *American Journal of Orthodontics and Dentofacial Orthopedics* destacaram-se com o maior número de publicações e citações (11 artigos, 532 e 461 citações, respectivamente). O autor com maior número de citações foi C.A. Feldens (n = 256). “*Malocclusion*” foi a palavra-chave mais utilizada (n = 39). As instituições mais produtivas foram Universidade Federal de Minas Gerais (12 artigos), Universidade Estadual de Campinas (11 artigos) e Universidade Federal de Santa Maria (8 artigos).

**Conclusão:**

Predominaram estudos transversais conduzidos por autores brasileiros examinando o impacto da má oclusão na qualidade de vida relacionada à saúde bucal. São necessários delineamentos mais diversos, maior representatividade geográfica e abordagens longitudinais para fortalecer as evidências sobre os efeitos biopsicossociais da má oclusão.

## INTRODUCTION

The biopsychosocial model conceptualizes health as an interaction between biological, psychological, and social dimensions, extending beyond a purely biomedical perspective.^1^ In Dentistry, this approach emphasizes patient-centered care and considers subjective experiences and social contexts affecting oral health outcomes.^2^ ^,^ ^3^ By integrating clinical, psychosocial and environmental factors, it provides a comprehensive framework for understanding the broader impacts of oral disorders.^4^

Malocclusion should not be considered solely a morphological issue but as a condition influencing multiple aspects of well-being, particularly during childhood and adolescence.^5^ ^-^ ^8^ Oral health is essential for functions like speaking, chewing, and swallowing, as well as psychological, emotional, and social well-being.^9^ Conditions such as malocclusion may cause functional and esthetic issues,^5^ impair oral health-related quality of life,^6^ reduce self-esteem due to embarrassment when smiling and speaking,^7^ and contribute to bullying during school age, when appearance and social interaction are critical to emotional and social development.^8^ ^,^ ^10^

The global prevalence of malocclusion in children and adolescents is 56%, with no significant sex differences.^11^ Regarding the epidemiological distribution among continents, the highest prevalence occurs in African countries (58%), followed by Europe (71%), the Americas (53%), and Asia (48%), with no data for Oceania.^11^ In Brazil, anterior open bite and posterior crossbite occur more frequently than in other regions.^12^

Systematic reviews and meta-analyses critically synthesize evidence to answer clinical questions.^13^ Bibliometric analyses, in contrast, examine scientific production, identifying patterns, trends, collaboration networks, and theoretical influences.^14^ Recognizing the most cited articles in dentistry helps guide research, education, and services.^15^

Understanding bibliometric indices related to the biopsychosocial impact of malocclusion allows researchers to map the current state of research and informs new studies aimed at improving quality of life, self-esteem, and reducing school-related psychosocial problems. This study analyzed the characteristics of the 100 most cited articles on the biopsychosocial impact of malocclusion on children and adolescents in the school environment.

## METHODS

This bibliometric review followed the guideline for reporting bibliometric reviews of the biomedical literature (BIBLIO).^16^

### INFORMATION SOURCES

The electronic search was conducted in the Web of Science Core Collection (WoS-CC) due to its broad interdisciplinary coverage and comprehensive metadata, which are well suited for bibliometric analyses. The use of a single database was intended to minimize the risk of duplicate records and reduce potential human error during data extraction and screening.

### SEARCH STRATEGY AND TIME PERIOD

Two independent examiners conducted a comprehensive search in the WoS-CC database in March 2025, using the following strategy: “Malocclusion” OR “Malocclusions” OR “Dental Malocclusion” (All Fields) AND “Models, Biopsychosocial” OR “Biopsychosocial Model” OR “Bullying” OR “Absenteeism” OR “Academic Achievement” OR “Academic Performance” OR “School Performance” OR “Educational Measurement” OR “Educational Test Score” OR “School Dropout” OR “Quality of Life” OR “Oral Health Related Quality of Life” OR “Self Concept” OR “Self Image” OR “Self Perception” OR “Self Esteem” OR “Emotional Intelligence” OR “Personal Satisfaction” (All Fields). No filters were applied during the search, and there were no restrictions for publication language or year.

### ELIGIBILITY CRITERIA AND DATA REFINEMENT

The identified results were organized in descending order of citations based on the data from the WoS-CC database. Two independent and previously calibrated reviewers (M.B.O. and D.C.F.) selected the articles by analyzing titles and abstracts, followed by the full texts. The inclusion criteria were articles on the biopsychosocial impact of malocclusion on school-aged children and adolescents. For the purposes of this bibliometric analysis, school-aged children and adolescents were defined as individuals enrolled in primary or secondary education, typically aged between 6 and 18 years. Editorials, conference papers, comments and letters to the editor, studies with preschool children and adults, questionnaire validations, research outside the school environment, and studies with children and/or adolescents wearing orthodontic appliances during the investigation were excluded. The articles meeting all eligibility criteria were included, and if the reviewers disagreed, a third party (L.R.P.) was consulted to resolve the conflict.

After selecting the 100 most cited articles, .txt and Excel files were exported from the WoS-CC database. The following information was extracted from each article: title, year of publication, authorship, number of citations, citation mean per year (calculated as the total number of citations divided by the number of years since publication until 2025), research center or affiliated institution/university, country and continent, study design, journal name, Journal Citation Reports Impact Factor (JCR^®^ IF 2024), and keywords. Two reviewers (M.B.O. and D.C.F.) independently evaluated the data to minimize errors. Country and continent information was determined based on the corresponding author’s affiliation. Study designs were classified as cross-sectional, case-control, and longitudinal.

### FULL-TEXT REVIEW AND CONTENT ANALYSIS

A full-text review and a content analysis were conducted to extract methodological and thematic information from the selected articles. The selected articles were fully analyzed to identify information about study sample, age group, sample size, malocclusion diagnosis indices, biopsychosocial outcomes, assessment instruments, and impact of malocclusion. Biopsychosocial outcomes were operationally classified into psychological and social domains. Psychological outcomes included oral health-related quality of life (OHRQoL), self-esteem, self-concept, self-image, emotional well-being, and personal satisfaction. Social outcomes comprised bullying experiences, peer relationships, social interaction difficulties, school absenteeism, and perceived social impact related to dental appearance.

### QUALITY ASSESSMENT

No formal methodological quality assessment was conducted, as this procedure is not applicable to the scope of the present study, in which the primary selection criterion was the impact of the articles in the scientific literature, measured by citation counts.

### DATA ANALYSIS AND VISUALIZATION

A graphical bibliometric network was generated using the Visualization of Similarities Viewer software (VOSviewer, version 1.6.17.0, Netherlands). The relationships between authors, institutions, and keywords were organized in clusters. The words linked to larger clusters and sources were more frequent, while those associated with smaller clusters and sources were less recurrent. The connections among clusters were represented by lines, portraying their interactions. Power BI (Microsoft, Redmond, Washington, USA) software was used to explore the relationship of the top 10 most cited regarding authorship, keywords, affiliations, and journal. The MapChart tool (https://mapchart.net/) illustrated the global distribution of the selected articles. 

### STATISTICAL ANALYSIS

A Poisson regression examined the association between the total number of citations from the articles and the independent variables. The articles were categorized according to the origin continent of the corresponding author (Africa, Asia, Europe, Oceania, North America, and South America), publication period (1996-2015 and 2016-2024), sample size (higher or lower than 597), and journal’s impact factor (higher or lower than 2.7) by the classification of the JCR^®^ IF 2024. The cutoff point for the sample size and impact factor variables was based on the median of all included studies. The analysis used R software for Windows, version 4.3.3. The findings were reported as rate ratios (RR) and respective confidence intervals (CI) at a 5% significance level (p < 0.05).

## RESULTS

A total of 1,297 records were identified and ranked in descending order of citation counts based on the WoS-CC database. After screening titles and abstracts, 1,146 articles were excluded for not meeting the eligibility criteria. A total of 151 articles underwent full-text review, with the 100 most-cited papers ultimately included in the study. Table 1 presents these articles ranked by citation count. 

**Table 1:** Top 100 most-cited papers on the biopsychosocial impact of malocclusion in school-aged children and adolescents.

Rank	Title	Year of Publication	Authorship	Web of Science - Core Collection (Mean per year)	Institution	Country	Continent	Study Design	Journal (JCR® IF 2024)	Keywords
1	Oral health-related quality of life of children by dental caries and fluorosis experience	2007	Do LG, Spencer A^19^	148 (7.79)	University of Adelaide	Australia	Oceania	Cross-sectional	Journal of Public Health Dentistry (1.5)	Oral health-related quality of life; children; fluorosis; caries
2	Perceived aesthetic impact of malocclusion and oral self-perceptions in 14-15-year-old Asian and Caucasian children in Greater Manchester	2000	Mandall NA, McCord JF, Blinkhorn AS, Worthington HV, O'Brien KD^20^	148 (5.69)	University of Manchester	The United Kingdom	Europe	Cross-sectional	European Journal of Orthodontics (2.7)	n.a.
3	Malocclusion: Esthetic impact and quality of life among Brazilian schoolchildren	2006	Marques LS, Ramos-Jorge ML, Paiva SM, Pordeus, IA^21^	127 (6.35)	Universidade Federal de Minas Gerais	Brazil	South America	Cross-sectional	American Journal of Orthodontics and Dentofacial Orthopedics (3.0)	n.a.
4	Psychosocial Impact of Dental Esthetics on Quality of Life in Adolescents Association with Malocclusion, Self-Image, and Oral Health-Related Issues	2009	De Paula DF, Santos NCM, Da Silva ET, Nunes MD, Leles CR^22^	124 (7.29)	Universidade Federal de Goiás	Brazil	South America	Cross-sectional	Angle Orthodontist (3.2)	Dental esthetics; malocclusion; adolescents
5	The influence of oral health conditions, socioeconomic status and home environment factors on schoolchildren's self-perception of quality of life	2012	Paula JS, Leite ICG, Almeida AB, Ambrosano GMB, Pereira AC, Mialhe FL^23^	117 (8.36)	Universidade Estadual de Campinas	Brazil	South America	Cross-sectional	Health and Quality of Life Outcomes (3.4)	n.a.
6	Disparities in oral health-related quality of life in a population of Canadian children	2007	Locker D^24^	110 (5.79)	University of Toronto	Canada	North America	Cross-sectional	Community Dentistry and Oral Epidemiology (2.1)	Children; disparities; oral health-related quality of life; socioeconomic status
7	Impact of Dental Disorders and its Influence on Self Esteem Levels among Adolescents	2017	Kaur P, Singh S, Mathur A, Makkar DK, Aggarwal VP, Batra M, Sharma A, Goyal N^25^	80 (8.89)	Surendera Dental College and Research Institute	India	Asia	Cross-sectional	Journal of Clinical and Diagnostic Research (0.2)	Dental caries; malocclusion; tooth loss
8	Bullying among Jordanian schoolchildren, its effects on school performance, and the contribution of general physical and dentofacial features	2013	Al-Bitar ZB, Al-Omari IK, Sonbol HN, Al-Ahmad HT, Cunningham SJ^26^	78 (6)	University of Jordan	Jordan	Asia	Cross-sectional	American Journal of Orthodontics and Dentofacial Orthopedics (3.0)	n.a.
9	Malocclusion and oral health-related quality of life in Brazilian school children: A population-based study	2013	Sardenberg F, Martins MT, Bendo CB, Pordeus IA, Paiva SM, Auad SM, Vale MP^27^	78 (6)	Universidade Federal de Minas Gerais	Brazil	South America	Cross-sectional	Angle Orthodontist (3.2)	Oral health-related quality of life; malocclusion; children
10	The effect of malocclusion and self-perceived aesthetics on the self-esteem of a sample of Jordanian adolescents	2010	Badran SA^28^	78 (4.88)	University of Jordan	Jordan	Asia	Cross-sectional	European Journal of Orthodontics (2.7)	n.a.
11	Orthodontic concern among 11-year-old children and their parents compared with orthodontic treatment need assessed by Index of Orthodontic Treatment Need	1996	Birkeland K, Boe OE, Wisth PJ^29^	77 (2.57)	University of Bergen	Norway	Europe	Cross-sectional	American Journal of Orthodontics and Dentofacial Orthopedics (3.0)	n.a.
12	Orthodontic treatment need for adolescents in the Campania region: the malocclusion impact on self-concept	2014	Perillo L, Esposito M, Caprioglio A, Attanasio S, Santini AC, Carotenuto M^30^	76 (6.33)	Second University of Naples	Italy	Europe	Cross-sectional	Patient Preference and Adherence (2.0)	Dental malocclusion; self-concept; adolescents; crossbite; dental crowding
13	Impact of molar-incisor hypomineralization on oral health-related quality of life in schoolchildren	2016	Dantas-Neta NB, Moura LDAD, Cruz PF, Moura MS, Paiva SM, Martins CC, Lima MDM^31^	74 (7.4)	Universidade Federal do Piauí	Brazil	South America	Cross-sectional	Brazilian Oral Research (1.3)	Quality of Life; dental enamel hypoplasia; oral health; tooth demineralization
14	Malocclusion impacts adolescents' oral health-related quality of life	2013	Scapini A, Feldens CA, Ardenghi TM, Kramer PF^32^	70 (5.38)	Universidade Luterana do Brasil	Brazil	South America	Cross-sectional	Angle Orthodontist (3.2)	Oral health-related quality of life; malocclusion; adolescent
15	Oral health-related quality of life of 11-and 12-year-old public school children in Rio de Janeiro	2011	Castro RDL, Portela MC, Leão AT, Vasconcellos MTL^33^	62 (4.13)	National Institute of Cardiology	Brazil	South America	Cross-sectional	Community Dentistry and Oral Epidemiology (2.1)	Oral health; pediatric dentistry; quality of life
16	Impact of molar incisor hypomineralization on quality of life in children with early mixed dentition: A hierarchical approach	2019	Portella PD, Menoncin BLV, Souza JF, Menezes JVNB, Fraiz FC, Assunçao LRD^34^	58 (8.29)	Universidade Federal do Paraná	Brazil	South America	Cross-sectional	International Journal of Paediatric Dentistry (1.9)	Dental enamel hypoplasia; oral health; quality of life; tooth demineralization
17	Clarifying the Impact of Untreated and Treated Dental Caries on Oral Health-Related Quality of Life among Adolescents	2016	Feldens CA, Ardenghi TM, Dullius AID, Vargas-Ferreira F, Hernandez PAG, Kranrier PF^35^	58 (5.8)	Universidade Luterana do Brasil	Brazil	South America	Cross-sectional	Caries Research (2.6)	Adolescent; dental caries; epidemiology; oral health; quality of life
18	Oral health-related quality of life of schoolchildren: impact of clinical and psychosocial variables	2015	Schuch HS, Costa FD, Torriani DD, Demarco FF, Goettems ML^36^	56 (5.09)	Universidade Federal de Pelotas	Brazil	South America	Cross-sectional	International Journal of Paediatric Dentistry (1.9)	n.a.
19	Determinants of children's oral-health-related quality of life over time	2014	Gururatana O, Baker SR, Robinson PG^37^	55 (4.58)	Sirindhorn College of Public Health Chonburi	Thailand	Asia	Longitudinal	Community Dentistry and Oral Epidemiology (2.1)	Children; dental coping beliefs; oral health; quality of life; sense of coherence; socioeconomic status
20	Psychosocial impact of malocclusion in Spanish adolescents	2013	Bellot-Arcís C, Montiel-Company JM, Almerich-Silla JM^38^	55 (4.23)	University of Valencia	Spain	Europe	Cross-sectional	Korean Journal of Orthodontics (2.3)	Public health; epidemiology
21	Oral Health-Related Quality of Life and Traumatic Dental Injuries in Young Permanent Incisors in Brazilian Schoolchildren: A Multilevel Approach	2015	Freire-Maia FB, Auad SM, Abreu MHNG, Sardenberg F, Martins MT, Paiva SM, Pordeus IA, Vale MP^39^	50 (4.55)	Universidade Federal de Minas Gerais	Brazil	South America	Cross-sectional	Plos One (2.6)	n.a.
22	Impact of bullying due to dentofacial features on oral health-related quality of life	2014	Al-Omari IK, Al-Bitar ZB, Sonbol HN, Al-Ahmad HT, Cunningham SJ, Al-Omiri M^40^	50 (4.17)	University of Jordan	Jordan	Asia	Cross-sectional	American Journal of Orthodontics and Dentofacial Orthopedics (3.0)	n.a.
23	Relationships between dental appearance, self-esteem, socio-economic status, and oral health-related quality of life in UK schoolchildren: A 3-year cohort study	2015	Benson PE, Da'as T, Johal A, Mandall NA, Williams AC, Baker SR, Marshman Z^41^	48 (4.36)	University of Sheffield	The United Kingdom	Europe	Longitudinal	European Journal of Orthodontics (2.7)	n.a.
24	Influence of quality of life, self-perception, and self-esteem on orthodontic treatment need	2017	Dos Santos PR, Meneghim MC, Ambrosano GMB, Vedovello M, Vedovello SAS^42^	46 (5.11)	Centro Universitário Hermínio Ometto	Brazil	South America	Cross-sectional	American Journal of Orthodontics and Dentofacial Orthopedics (3.0)	n.a.
25	Oral health-related quality of life and traumatic dental injuries in Brazilian adolescents	2014	Bendo CB, Paiva SM, Varni JW, Vale MP^43^	45 (3.75)	Universidade Federal de Minas Gerais	Brazil	South America	Case-control	Community Dentistry and Oral Epidemiology (2.1)	Epidemiology; injuries; oral health; pediatric dentistry; quality of life
26	The impact of malocclusion on adolescents' dissatisfaction with dental appearance and oral functions	2012	Tessarollo FR, Feldens CA, Closs LQ^44^	44 (3.14)	Universidade Luterana do Brasil	Brazil	South America	Cross-sectional	Angle Orthodontist (3.2)	Epidemiology; malocclusion; personal satisfaction; smiling; mastication; speech
27	Impacts on daily performances attributed to malocclusions using the condition-specific feature of the oral impacts on daily performances index	2008	Bernabé E, Tsakos G, De Oliveira CM, Sheiham A^45^	44 (2.44)	University College London	The United Kingdom	Europe	Cross-sectional	Angle Orthodontist (3.2)	Oral impacts; condition-specific impacts; malocclusion; orthodontic need; adolescents
28	The relationship between normative orthodontic treatment need and measures of consumer perception	2001	Mandall NA, Wright J, Conboy FM, O'Brien KD^46^	44 (1.76)	University Dental Hospital of Manchester	The United Kingdom	Europe	Cross-sectional	Community Dental Health (1.0)	Index of orthodontic treatment need; malocclusion; oral aesthetic impact; psycho-social impact; self esteem
29	Clinical status in adolescents: is its impact on oral health-related quality of life influenced by psychological characteristics?	2013	Foster Page LA, Thomson WM, Ukra A, Baker SR^47^	43 (3.31)	University of Otago	New Zealand	Oceania	Cross-sectional	European Journal of Oral Sciences (1.8)	Adolescents; child perception questionnaire; psychological characteristics; quality of life
30	Impact of traumatic dental injuries on the quality of life of schoolchildren	2012	Traebert J, Lacerda JT, Page LAF, Thomson WM, Bortoluzzi MC^48^	43 (3.07)	Universidade do Sul de Santa Catarina	Brazil	South America	Cross-sectional	Dental Traumatology (3.1)	Oral health-related quality of life; traumatic dental injuries; dental trauma; schoolchildren
31	Occlusal traits and perception of orthodontic need in eighth grade students	1998	Sheats RD, McGorray SP, Keeling SD, Wheeler TT, King GJ^49^	43 (1.54)	Mayo Clinic	The United States	North America	Cross-sectional	Angle Orthodontist (3.2)	Malocclusion; self-concept; perceived need; esthetics
32	Socioeconomic inequalities in oral health-related quality of life in adolescents: a cohort study	2019	Sfreddo CS, Moreira CHC, Nicolau B, Ortiz FR, Ardenghi TM^50^	41 (5.86)	Universidade Federal de Santa Maria	Brazil	South America	Longitudinal	Quality of Life Research (2.7)	Socioeconomic factors; health inequalities; quality of life; oral health; observational study
33	Impact of malocclusion and dentofacial anomalies on the prevalence and severity of dental caries among adolescents	2015	Feldens CA, Dullius AIDS, Kramer PF, Scapini A, Busato ALS, Vargas-Ferreira F^51^	41 (3.73)	Universidade Luterana do Brasil	Brazil	South America	Cross-sectional	Angle Orthodontist (3.2)	Malocclusion; adolescent; dental caries
34	Factors influencing adolescents' oral health-related quality of life (OHRQoL)	2013	Foster Page LA, Thomson WM, Ukra A, Farella M^52^	41 (3.15)	University of Otago	New Zealand	Oceania	Cross-sectional	International Journal of Paediatric Dentistry (1.9)	n.a.
35	The impact of orthodontic treatment on the quality of life in adolescents: a case-control study	2008	Bernabé E, Sheiham A, Tsakos G, De Oliveira CM^53^	40 (2.22)	University College London	The United Kingdom	Europe	Case-control	European Journal of Orthodontics (2.7)	n.a.
36	Desire for orthodontic treatment and associated factors among adolescents in southern Brazil	2015	Feldens CA, Nakamura EK, Tessarollo FR, Closs LQ^54^	38 (3.45)	Universidade Luterana do Brasil	Brazil	South America	Cross-sectional	Angle Orthodontist (3.2)	Orthodontics; malocclusion; desire; motivation
37	The factors that influence oral health-related quality of life in 15-year-old children	2018	Sun L, Wong HM, McGrath CPJ^55^	35 (4.38)	University of Hong Kong	China	Asia	Cross-sectional	Health and Quality of Life Outcomes (3.4)	Oral health-related quality of life; periodontal status; caries; malocclusion; sociodemographic factors
38	Association between malocclusion and the contextual factors of quality of life and socioeconomic status	2016	Vedovello SAS, Ambrosano GMB, Pereira AC, Valdrighi HC, Vedovello M, Meneghim MC^56^	35 (3.5)	Centro Universitário Hermínio Ometto	Brazil	South America	Cross-sectional	American Journal of Orthodontics and Dentofacial Orthopedics (3.0)	n.a.
39	Objective, Subjective, and Self-Assessment of Preadolescent Orthodontic Treatment Need - A Function of Age, Gender, and Ethnic/Racial Background?	2009	Christopherson EA, Briskie D, Inglehart MR^57^	35 (2.06)	University of Michigan System	The United States	North America	Cross-sectional	Journal of Public Health Dentistry (1.5)	Orthodontic treatment need; malocclusion; children; adolescents; self-perceptions; oral health-related quality of life
40	Factors Associated with Oral Health-related Quality of Life in Children and Preadolescents: A Cross-sectional Study	2016	Barbosa TD, Gaviao MBD, Castelo PM, Leme MS^58^	34 (3.4)	Universidade Estadual de Campinas	Brazil	South America	Cross-sectional	Oral Health & Preventive Dentistry (1.4)	Adolescent; child; oral health-related quality of life; psychological factors
41	Oral-health-related quality of life in schoolchildren in an endemic fluorosis area of Mexico	2011	Aguilar-Díaz FC, Irigoyen-Camacho ME, Borges-Yáñez SA^59^	34 (2.27)	Universidad Nacional Autonoma de Mexico	Mexico	North America	Cross-sectional	Quality of Life Research (2.7)	Quality of life; dental fluorosis; caries; malocclusion; schoolchildren
42	Effect of anterior teeth display during smiling on the self-perceived impacts of malocclusion in adolescents	2011	Paula DF, Silva ET, Campos ACV, Nuñez MO, Leles CR^60^	34 (2.27)	Universidade Federal de Goiás	Brazil	South America	Cross-sectional	Angle Orthodontist (3.2)	Dental esthetics; anterior teeth display; malocclusion; adolescents
43	Negative effect of malocclusion on the emotional and social well-being of Brazilian adolescents: a population-based study	2017	Bittencourt JM, Martins LP, Bendo CB, Vale MP, Paiva SM^5^	33 (3.67)	Universidade Federal de Minas Gerais	Brazil	South America	Cross-sectional	European Journal of Orthodontics (2.7)	n.a.
44	Dental caries remains as the main oral condition with the greatest impact on children's quality of life	2017	Martins MT, Sardenberg F, Bendo CB, Abreu MH, Vale MP, Paiva SM, Pordeus IA^61^	33 (3.67)	Universidade Federal de Minas Gerais	Brazil	South America	Case-control	Plos One (2.6)	n.a.
45	Aesthetic impact of malocclusion in the daily living of Brazilian adolescents	2009	Marques LS, Filogônio CA, Filogônio CB, Pereira LJ, Pordeus IA, Paiva SM, Ramos-Jorge ML^62^	33 (1.94)	Universidade Vale do Rio Verde	Brazil	South America	Cross-sectional	Journal of Orthodontics (1.5)	Aesthetics; quality of life; malocclusion; adolescents
46	The factors that influence the oral health-related quality of life in 12-year-old children: baseline study of a longitudinal research	2017	Sun L, Wong HM, McGratSh CPJ^63^	32 (3.56)	University of Hong Kong	China	Asia	Cross-sectional	Health and Quality of Life Outcomes (3.4)	Oral health-related quality of life; periodontal status; caries; malocclusion; sociodemographic factors; baseline study
47	Association of malocclusion, happiness, and oral health-related quality of life (OHRQoL) in schoolchildren	2016	Da Rosa GN, Del Fabro JP, Tomazoni F, Tuchtenhagen S, Alves LS, Ardenghi TM^64^	31 (3.1)	Universidade Federal de Santa Maria	Brazil	South America	Cross-sectional	Journal of Public Health Dentistry (1.5)	Malocclusion; quality of life; happiness; cross-sectional studies
48	Exploring the association between oral health problems and oral health-related quality of life in Peruvian 11-to 14-year-old children	2016	Pulache J, Abanto J, Oliveira LB, Bönecker M, Porras JC^65^	31 (3.1)	Universidade de São Paulo	Brazil	South America	Cross-sectional	International Journal of Paediatric Dentistry (1.9)	n.a.
49	Associations between oral health-related quality of life and emotional statuses in children and preadolescents	2012	Barbosa TS, Castelo PM, Leme MS, Gavião MBD^66^	31 (2.21)	Universidade Estadual de Campinas	Brazil	South America	Cross-sectional	Oral diseases (2.9)	Anxiety; child; depression; oral health-related quality of life; preadolescent; salivary cortisol
50	Malocclusion: Social, Functional and Emotional Influence on Children	2012	Martins-Júnior PA , Marques LS, Ramos-Jorge ML^67^	31 (2.21)	Universidade Federal dos Vales do Jequitinhonha e Mucuri	Brazil	South America	Cross-sectional	Journal of Clinical Pediatric Dentistry (2.2)	Oral health-related quality of life; malocclusion; children
51	Differences between normative criteria and self-perception in the assessment of malocclusion	2002	Peres KG, Traebert ESD, Marcenes W^68^	31 (1.29)	Universidade do Sul de Santa Catarina	Brazil	South America	Cross-sectional	Revista de Saude Publica (2.1)	Malocclusion, therapy; perception; cross-sectional studies; malocclusion, epidemiology; prevalence; orthodontics, treatment
52	Assessment of oral health-related quality of life in Nigerian children using the Child Perceptions Questionnaire (CPQ 11-14)	2011	Kolawole KA, Otuyemi OD, Oluwadaisi AM^69^	28 (1.87)	Obafemi Awolowo University	Nigeria	Africa	Cross-sectional	European Journal of Paediatric Dentistry (2.7)	Children; Oral health; Quality of life
53	Impact of malocclusion on oral health-related quality of life among schoolchildren	2018	de Araújo Guimarães SP, Jorge KO, Fontes MJF, Ramos-Jorge ML, Araújo CTP, Ferreira EF, Melgaço CA, Zarzar PM^70^	26 (3.25)	Universidade Federal dos Vales do Jequitinhonha e Mucuri	Brazil	South America	Cross-sectional	Brazilian Oral Research (1.3)	Quality of Life; malocclusion; students; adolescent
54	Dental caries are more likely to impact on children's quality of life than malocclusion or traumatic dental injuries	2018	Martins MT, Sardenberg F, Bendo CB, Vale MP, Paiva SM, Pordeus IA^71^	26 (3.25)	Universidade Federal de Minas Gerais	Brazil	South America	Cross-sectional	European Journal of Paediatric Dentistry (2.7)	Children; dental caries; malocclusion; quality of life; traumatic dental injuries
55	The influence of normative and subjective oral health status on schoolchildren's happiness	2015	Tuchtenhagen S, Bresolin CR, Tomazoni F, Da Rosa GN, Del Fabro JP, Mendes FM, Antunes JLF, Ardenghi TM^72^	25 (2.27)	Universidade Federal de Santa Maria	Brazil	South America	Cross-sectional	BMC Oral Health (3.1)	Happiness; child; oral health; quality of life
56	An assessment of relationship between self-esteem, orthodontic concern, and Dental Aesthetic Index (DAI) scores among secondary school students in Ibadan, Nigeria	2003	Onyeaso CO^73^	25 (1.09)	University of Ibadan	Nigeria	Africa	Cross-sectional	International Dental Journal (3.7)	Orthodontic assessment; Dental Aesthetic Index (DAI); appearance; self-esteem; Nigeria
57	School environment and individual factors influence oral health related quality of life in Brazilian children	2018	Machry RV, Knorst JK, Tomazoni F, Ardenghi TM^74^	24 (3)	Universidade Federal de Santa Maria	Brazil	South America	Cross-sectional	Brazilian Oral Research (1.3)	Child; quality of life; oral health
58	Associations between Malocclusion and Oral Health-Related Quality of Life among Mongolian Adolescents	2017	Araki M, Yasuda Y, Ogawa T, Tumurkhuu T, Ganburged G, Bazar A, Fujiwara T, Moriyama K^75^	24 (2.67)	Tokyo Medical and Dental University	Japan	Asia	Cross-sectional	International Journal of Environmental Research and Public Health (4.614)	Child perceptions questionnaire; malocclusion; Mongolia; oral health-related quality of life
59	Impact of Malocclusions on the Oral Health-Related Quality of Life of Early Adolescents in Ndola, Zambia	2018	Anthony SN, Zimba K, Subramanian B^76^	22 (2.75)	Copperbelt University	Zambia	Africa	Cross-sectional	International Journal of Dentistry (2.2)	n.a.
60	Condition-specific sociodental impacts attributed to different anterior occlusal traits in Brazilian adolescents	2007	Bernabé E, De Oliveira CM, Sheiham A^77^	22 (1.16)	University College London	The United Kingdom	Europe	Cross-sectional	European Journal of Oral Sciences (1.8)	Adolescents; malocclusion; quality of life; social impacts
61	Impact of Dental Trauma on Quality of Life among 11-14 Years Schoolchildren	2017	El-Kalla IH, Shalan HM, Bakr RA^78^	21 (2.33)	Mansoura University	Egypt	Africa	Cross-sectional	Contemporary Clinical Dentistry (1.1)	Dental trauma; malocclusion; quality of life
62	Oral disorders associated with the experience of verbal bullying among Brazilian school-aged children A case-control study	2020	Duarte-Rodrigues L, Ramos-Jorge ML, Alves-Duarte AC, Fonseca-Silva T, Flores-Mir C, Marques LS^79^	20 (3.33)	Universidade Federal dos Vales do Jequitinhonha e Mucuri	Brazil	South America	Case-control	Journal of the American Dental Association (3.5)	Oral health; bullying; quality of life; malocclusion
63	Effect of malocclusion severity on oral health related quality of life in Malay adolescents	2021	Elyaskhil M, Shafai NAA, Mokhtar N^80^	18 (3.6)	Universiti Sains Malaysia	Malaysia	Asia	Cross-sectional	Health and Quality of Life Outcomes (3.4)	Malocclusion severity; oral health related quality of life; Malay adolescents
64	Impact of Malocclusion on the Quality of Life of Brazilian Adolescents: A Population-Based Study	2016	Silva LFGE, Thomaz EBAF, Freitas HV, Pereira ALP, Ribeiro CCC, Alves CMC^81^	18 (1.8)	Universidade Ceuma	Brazil	South America	Cross-sectional	Plos One (2.6)	n.a.
65	Negative self-perception of smile associated with malocclusions among Brazilian adolescents	2013	Moura C, Cavalcanti AL, Gusmão ES, Soares RDC, Moura FTC, Santillo PMH^82^	18 (1.38)	Universidade Federal de Campina Grande	Brazil	South America	Cross-sectional	European Journal of Orthodontics (2.7)	n.a.
66	Self-perception and malocclusion and their relation to oral appearance and function	2011	Peres SHDS, Goya S, Cortellazzi KL, Ambrosano GMB, Meneghim MC, Pereira AC^83^	17 (1.13)	Universidade de São Paulo	Brazil	South America	Cross-sectional	Ciencia & Saude Coletiva (1.2)	Malocclusion; adolescent health; self-concept; oral health
67	The Relationship of Malocclusion as Assessed by the Dental Aesthetic Index (DAI) with Perceptions of Aesthetics, Function, Speech and Treatment Needs Among 14- to 15-year-old Schoolchildren of Bangalore, India	2010	Nagarajan S, Pushpanjali K^84^	17 (1.06)	SVS Institute of Dental Sciences	India	Asia	Cross-sectional	Oral Health & Preventive Dentistry (1.4)	Dental Aesthetic Index; malocclusion; subjective perceptions
68	Oral health and happiness in adolescents: A cohort study	2021	Tuchtenhagen S, Ortiz FR, Ardenghi TM, Antunes JLF^85^	15 (2.5)	Universidade Regional Integrada do Alto Uruguai e das Missões	Brazil	South America	Longitudinal	Community Dentistry and Oral Epidemiology (2.1)	Adolescent; cohort studies; happiness; oral health; quality of life
69	Malocclusion, Dental Caries and Oral Health-Related Quality of Life: A Comparison between Adolescent School Children in Urban and Rural Regions in Peru	2020	De Llano-Pérula MC, Ricse E, Fieuws S, Willems G, Orellana-Valvekens MF^86^	15 (2.5)	University Hospital Leuven	Belgium	Europe	Cross-sectional	International Journal of Environmental Research and Public Health (4.614)	Community-based study; occlusion; orthodontics; occlusal indices; caries prevalence; oral health related quality of life
70	Impact of treated and untreated traumatic dental injuries on oral health-related quality of life among 12-year-old schoolchildren in Amman	2019	Rajab LD, Abu Al Huda D^87^	15 (2.14)	University of Jordan	Jordan	Asia	Cross-sectional	Dental Traumatology (3.1)	Children; Jordan; quality of life; traumatic dental injuries
71	Correlation Between Oral Health and Child-OIDP Index in 12-and 15-Year-Old Children From Modinagar, India	2014	Basavaraj P, Sunil MK, Nagarajappa R, Ashish S, Ramesh G^88^	15 (1.25)	DJ College of Dental Sciences and Research	India	Asia	Cross-sectional	Asia-Pacific Journal of Public Health (0.9)	Child-Oral Impact on Daily Performance; dental caries; dentofacial anomalies; oral health-related quality of life; tooth injuries
72	Preadolescent orthodontic treatment need: Objective and subjective provider assessments and patient self-reports	2009	Christopherson EA, Briskie D, Inglehart MR^89^	15 (0.88)	University of Michigan	The United States	North America	Cross-sectional	American Journal of Orthodontics and Dentofacial Orthopedics (3.0)	n.a.
73	Esthetic impact of malocclusions in the anterior segment on children in the mixed dentition	2021	Nabarrette M, Brunheroto J, Dos Santos PR, Meneghim MC, Vedovello SAS^90^	14 (2.8)	Universidade Estadual de Campinas	Brazil	South America	Cross-sectional	American Journal of Orthodontics and Dentofacial Orthopedics (3.0)	n.a.
74	Malocclusion, facial and psychological predictors of quality of life in adolescents	2019	Dallé H, Vedovello SAS, Degan VV, De Godoi APT, Custódio W, De Menezes CC^91^	14 (2)	Centro Universitário Hermínio Ometto	Brazil	South America	Cross-sectional	Community Dental Health (1.0)	Quality of life; malocclusion; self-perception; facial profile
75	Orthodontic treatment need, self-esteem, and oral health-related quality of life among 12-yr-old schoolchildren	2019	Herkrath APCQ, Vettore MV, De Queiroz AC, Alves PLN, Leite SDC, Pereira JV, Rebelo MAB, Herkrath FJ^92^	14 (2)	University of Sheffield	The United Kingdom	Europe	Cross-sectional	European Journal of Oral Sciences (1.8)	Malocclusion; quality of life; self-concept
76	Factors related to the psychological impact of malocclusion in adolescents	2020	Iranzo-Cortés JE, Montiel-Company JM, Bellot-Arcis C, Almerich-Torres T, Acevedo-Atala C, Ortolá-Siscar JC, Almerich-Silla JM^93^	13 (2.17)	University of Valencia	Spain	Europe	Cross-sectional	Scientific Reports (3.9)	n.a.
77	A cohort study of factors that influence oral health-related quality of life from age 12 to 18 in Hong Kong	2020	Sun L, Wong HM, McGrath CPJ^94^	13 (2.17)	University of Hong Kong	China	Asia	Longitudinal	Health and Quality of Life Outcomes (3.4)	Oral health-related quality of life; periodontal status; caries; malocclusion; sociodemographic factors
78	Relationship Between Orthodontic Treatment Need and Oral Health-Related Quality of Life among 11-18-Year-Old Adolescents in Lithuania	2018	Kavaliauskienė A, Šidlauskas A, Zaborskis A^95^	13 (1.63)	Lithuanian University of Health Sciences	Lithuania	Europe	Cross-sectional	International Journal of Environmental Research and Public Health (4.614)	Oral health-related quality of life; child perception questionnaire; malocclusion; orthodontic treatment need; associations; adolescents; Lithuania
79	Impact of malocclusion on oral health-related quality of life in 10-14-year-old children of Mumbai, India	2016	Bhatia R, Winnier JJ, Mehta N^96^	13 (1.3)	D.Y. Patil University School of Dentistry	India	Asia	Cross-sectional	Contemporary Clinical Dentistry (1.1)	Child Perception Questionnaire 11-14; Index of Orthodontic Treatment Need; malocclusion; oral health-related quality of life
80	Association between Global Life Satisfaction and Self-Rated Oral Health Conditions among Adolescents in Lithuania	2017	Kavaliauskienė A, Šidlauskas A, Zaborskis A^97^	12 (1.33)	Lithuanian University of Health Sciences	Lithuania	Europe	Cross-sectional	International Journal of Environmental Research and Public Health (4.614)	Quality of life; life satisfaction; oral health; oral health-related quality of life; adolescents; child perceptions questionnaire
81	The role of parental rearing practices and family demographics on oral health-related quality of life in children	2017	Kumar S, Zimmer-Gembeck MJ, Kroon J, Lalloo R, Johnson NW^98^	12 (1.33)	Griffith University	Australia	Oceania	Cross-sectional	Quality of Life Research (2.7)	Oral health; quality of life; parental rearing practices; family structure; socioeconomic status
82	Comparison of Normative Methods and the Sociodental Approach to Assessing Orthodontic Treatment Needs in 12-year-old Schoolchildren	2013	Herkrath FJ, Rebelo MAB, Herkrath APCD, Vettore MV^99^	12 (0.92)	Universidade Federal do Rio de Janeiro	Brazil	South America	Cross-sectional	Oral Health & Preventive Dentistry (1.4)	Health services needs and demand; malocclusion; needs assessment; orthodontics; quality of life
83	Correlation between malocclusion and history of bullying in vulnerable adolescents	2022	Ramos ITM, Nabarrette M, Vedovello M, De Menezes CC, Meneghim MC, Vedovello SAS^100^	11 (2.75)	Centro Universitário Hermínio Ometto	Brazil	South America	Cross-sectional	Angle Orthodontist (3.2)	Bullying; malocclusion; epidemiology
84	How is orthodontic treatment need associated with perceived esthetic impact of malocclusion in adolescents?	2020	De Oliveira Meira ACL, Custodio W, Vedovello M, Borges TM, Meneghim MC, Santamaria M, Vedovello SAS^101^	11 (1.83)	Centro Universitário Hermínio Ometto	Brazil	South America	Cross-sectional	American Journal of Orthodontics and Dentofacial Orthopedics (3.0)	n.a.
85	Association between normative and self-perceived orthodontic treatment need among 12-to 15-year-old students in Shiraz, Iran	2010	Danaei SM, Salehi P^102^	11 (0.69)	Shiraz University of Medical Science	Iran	Asia	Cross-sectional	European Journal of Orthodontics (2.7)	n.a.
86	Malocclusion in Brazilian Schoolchildren: High Prevalence and Low Impact	2018	Traebert E, Martins LGT, Pereira KCR, Costa SXS, Lunardelli SE, Lunardelli AN, Traebert J^103^	10 (1.25)	Universidade do Sul de Santa Catarina	Brazil	South America	Cross-sectional	Oral Health & Preventive Dentistry (1.4)	Epidemiology; impact; malocclusion; oral health; schoolchildren
87	Influence of sense of coherence on adolescents' self-perceived dental aesthetics; a cross-sectional study	2017	Costa AC, Rodrigues FS, Da Fonte PP, Rosenblatt A, Innes NPT, Heimer MV^104^	10 (1.11)	University of Dundee	The United Kingdom	Europe	Cross-sectional	BMC Oral Health (3.1)	Sense of coherence; self-perception; dental aesthetics; adolescent
88	Incisor Molar Hypomineralization and Quality of Life: A Population-Based Study with Brazilian Schoolchildren	2021	Fernandes LHF, Laureano ICC, Farias L, Andrade NM, Forte FDS, Alencar CRB, Cavalcanti AL^105^	9 (1.8)	Universidade Estadual da Paraíba	Brazil	South America	Cross-sectional	International Journal of Dentistry (2.2)	n.a.
89	Oral health-related quality-of-life scores differ by socioeconomic status, mother's level of education, dental visits and severity of malocclusion in mixed dentition of eight-to-ten-year-old schoolchildren	2021	García Pérez AG, Pineda AEGA, Olivares HG^106^	8 (1.6)	Universidad Nacional Autonoma de Mexico	Mexico	North America	Cross-sectional	PeerJ (2.4)	Socioeconomic factors; mother's level of education; OHRQoL; schoolchildren; malocclusion
90	Impact of anterior occlusal conditions in the mixed dentition on oral health-related quality-of-life item levels: A multivariate analysis	2020	Vedovello SAS, De Carvalho ALM, De Azevedo LC, Dos Santos PR, Vedovello M, Meneghim MC^107^	8 (1.33)	Centro Universitário Hermínio Ometto	Brazil	South America	Cross-sectional	Angle Orthodontist (3.2)	Mixed dentition; quality of life; oral health; malocclusion
91	Relationships of Dental Caries and Malocclusion with Oral Health-Related Quality of Life in Lithuanian Adolescents Aged 15 to 18 Years: A Cross-Sectional Study	2020	Kavaliauskienė A, Šidlauskas A, Žemaitienė M, Slabšinskienė E, Zaborskis A^108^	8 (1.33)	Lithuanian University of Health Sciences	Lithuania	Europe	Cross-sectional	International Journal of Environmental Research and Public Health (4.614)	Oral health-related quality of life; child perceptions questionnaire; dental caries; malocclusion; adolescents; Lithuania
92	The effect of enamel fractures on oral health-related quality of life in adolescents	2020	Feldens CA, Senna RA, Vargas-Ferreira F, Braga VS, Feldens EG, Kramer PF^109^	7 (1)	Universidade Luterana do Brasil	Brazil	South America	Cross-sectional	Dental Traumatology (3.1)	Adolescent; quality of life; tooth injuries
93	Prevalence, Extent and Severity of the Psychosocial Impact of Dental Aesthetics among Malaysian Adolescents	2019	Hassan WNW, Yusof ZYM, Yuen SW, Tajudin ZM, Lokman N, Makhbul MZM^110^	7 (1)	University of Malaya	Malaysia	Asia	Cross-sectional	Sains Malaysiana (0.8)	Adolescent health; malocclusion; oral health care; oral health-related quality of life; orthodontics
94	Demographic and social inequalities in need for orthodontic treatment among schoolchildren in Lithuania	2010	Kavaliauskienė A, Sidlauskas A, Zaborskis A^111^	7 (0,44)	Lithuanian University of Health Sciences	Lithuania	Europe	Cross-sectional	Medicina-Lithuania (2.4)	Schoolchildren; survey; oral health; orthodontic anomalies; social factors; health inequalities
95	Orthodontic Treatment Complexity and Need with Associated Oral Health-Related Quality of Life in Nigerian Adolescents	2009	Onyeaso CO^112^	7 (0.41)	University of Port Harcourt	Nigeria	Africa	Cross-sectional	Oral Health & Preventive Dentistry (1.4)	Nigerian adolescents; oral health-related quality of life; orthodontic treatment complexity and need
96	Oral health-related quality of life determinants throughout adolescence: a cohort study in Brazil	2022	Ortiz FR, Emmanuelli B, De Campos AM, Ardenghi TM^113^	6 (1.5)	Universidade Federal de Santa Maria	Brazil	South America	Longitudinal	Quality of Life Research (2.7)	Oral health; quality of life; adolescents; longitudinal studies
97	Oral health-related quality of life of adolescents assessed with the Malocclusion Impact and Child Perceptions questionnaires	2021	Kolawole KA, Ayodele-Oja MM^114^	6 (1.2)	Obafemi Awolowo University	Nigeria	Africa	Cross-sectional	American Journal of Orthodontics and Dentofacial Orthopedics (3.0)	n.a.
98	Dental aesthetics and self-esteem in adolescents	2011	Mafla AC, Luna EG, Sánchez NR, Barrera DA, Muñoz GM^115^	6 (0.4)	Universidad Cooperativa de Colombia	Colombia	South America	Cross-sectional	Colombia Medica (1.3)	Aesthetics; Dental Aesthetic Index; malocclusion; self-esteem Rosenberg Scale; adolescent; socioeconomic status
99	Contextual and individual factors associated with traumatic dental injuries in deprived 12-year-old schoolchildren: A cohort study	2024	Bezerra EDN, Herkrath FJ, Vettore MV, Rebelo MAB, De Queiroz AC, Vieira JMR, Pereira JV, Freitas MOD, Herkrath APCD^17^	5 (2.5)	Universidade Federal de Amazonas	Brazil	South America	Longitudinal	Dental Traumatology (3.1)	Health social determinants; malocclusion; self-image; sense of coherence; traumatic dental injuries
100	Esthetic impact of maxillary midline diastema and mandibular crowding in children in the mixed dentition	2022	Carneiro DPA, Venezian GC, Valdrighi HC, Meneghim MC, Vedovello SAS^18^	5 (1.25)	Centro Universitário Herminio Ometto	Brazil	South America	Cross-sectional	American Journal of Orthodontics and Dentofacial Orthopedics (3.0)	n.a.

n.a.: not applicable; JCR^®^ IF: Journal Citation Report Impact Factor.

The 100 most-cited articles accumulated a total of 3,621 citations, ranging from 5^17^ ^,^ ^18^ to 148^19^ ^,^ ^20^ per article, with a mean of 36.21 citations. Six studies received more than 100 citations^19^ ^-^ ^24^ (Table 1). A total of 36 journals were represented. *The Angle Orthodontist* and the *American Journal of Orthodontics and Dentofacial Orthopedics* had the highest number of publications and citations (11 articles, 532 and 461 citations, respectively), followed by the *European Journal of Orthodontics* (7 articles, 374 citations) and *Community Dentistry and Oral Epidemiology* (5 articles, 287 citations) (Table 1).

**Figure 1:**
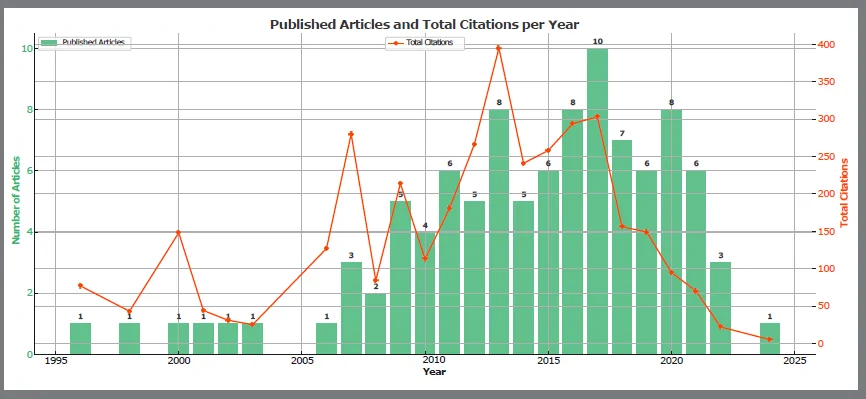
Annual distribution of publications and citations among the 100 most-cited articles.

The earliest article was published in 1996 (77 citations, average 2.57 citations per year), and the most recent in 2024 (5 citations, average 2.5 citations per year) (Table 1, Fig. 1). Most publications occurred between 2015 and 2019 (37 articles), with 2017 showing the highest number of publications (n = 10). Citation peaks occurred between 2010 and 2014 (n = 1,196), with 2013 contributing 395 citations (Fig. 1).

**Figure 2:**
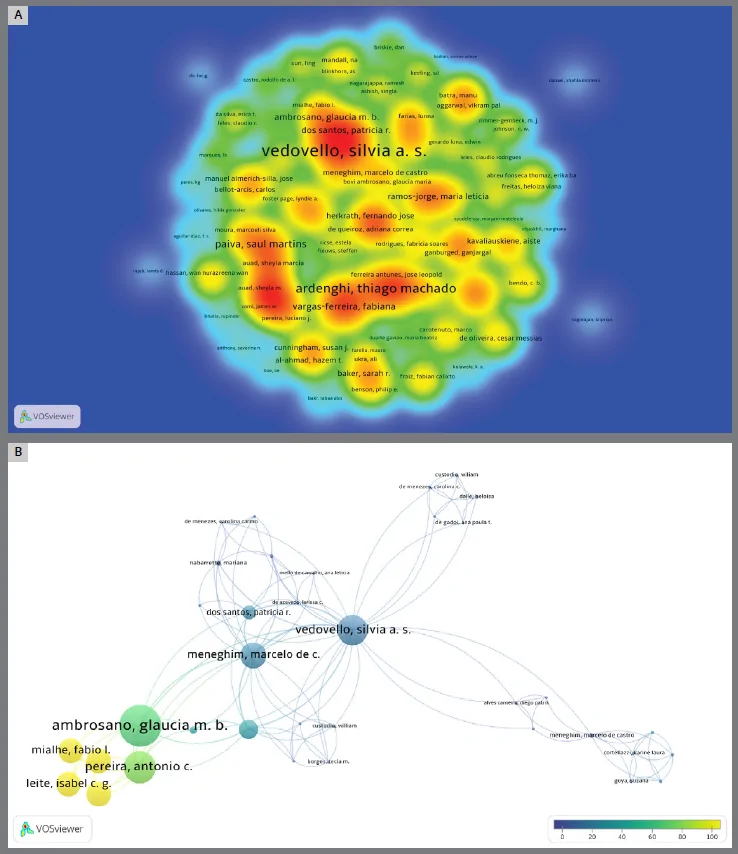
Co-authorship networks among the top 100 articles. **A**) Density map displaying 60 author clusters. Red indicates stronger total link strength between authors, whereas blue indicates weaker link strength. **B**) Overlay visualization highlighting the main clusters among the included studies. The scale bar represents average citation counts.

Co-authorship analysis identified 363 authors across 60 clusters, five of which exhibited strong connections, comprising 36 authors (Fig. 2A-B). The most prolific authors were S.M. Paiva (9 articles, 499 citations), T.M. Ardenghi (8 articles, 270 citations), M.C. Meneghim (8 articles, 147 citations), S.A.S. Vedovello (8 articles, 144 citations), and C.A. Feldens (6 articles, 258 citations), with S.M. Paiva having the highest total citations.

**Figure 3:**
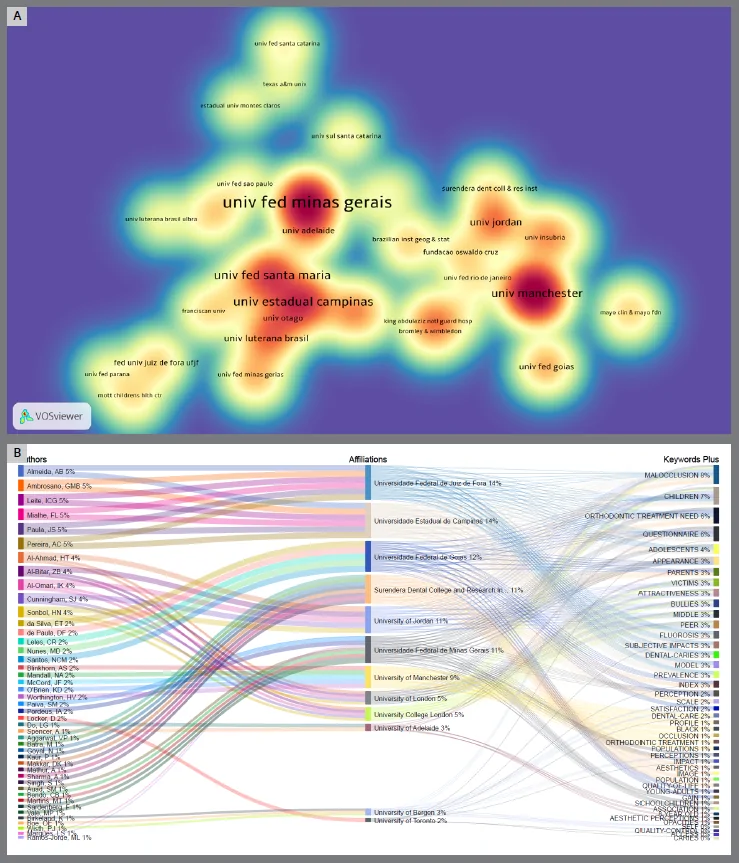
A) Institutional affiliations of contributing authors (institutions with ≥40 citations included). B) Sankey plot illustrating correlations among authors, affiliations, and Keywords Plus. Co-occurrence network of keywords appearing two or more times in the 50 most cited articles over time.

A total of 121 organizations were identified, with 51 meeting the analysis threshold (Fig. 3A). The Federal University of Minas Gerais (12 articles, 514 citations), the State University of Campinas (11 articles, 328 citations), and the Federal University of Santa Maria (8 articles, 304 citations) were the most productive institutions. Among the top 10 most-cited articles, the Federal University of Juiz de Fora and the State University of Campinas were most frequently represented across authors and Keywords Plus (14% each) (Fig. 3B).

**Figure 4:**
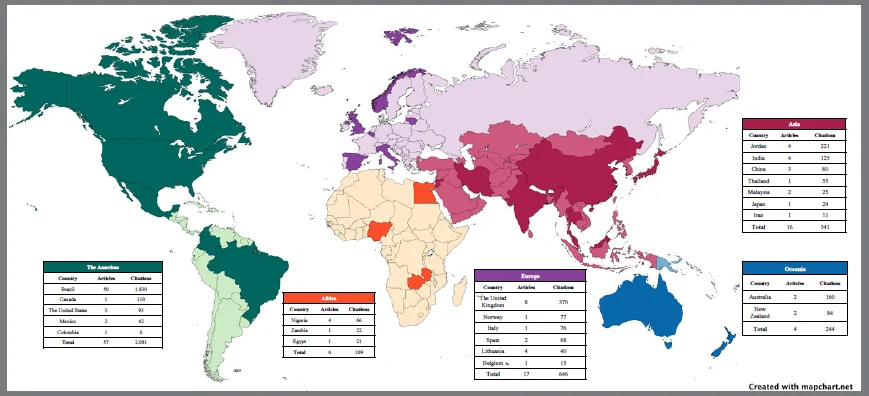
Global distribution of the 100 most-cited articles on the biopsychosocial impact of malocclusion in school-aged children and adolescents.

Corresponding authors came from 23 countries. Brazil led with 50 publications (1,830 citations), followed by the United Kingdom (8 articles, 370 citations), Jordan (4 articles, 221 citations), India (4 articles, 125 citations), Nigeria (4 articles, 66 citations), and Lithuania (4 articles, 40 citations). All continents contributed, with the Americas having the highest output (57 articles, 2,081 citations) (Fig. 4).

**Figure 5:**
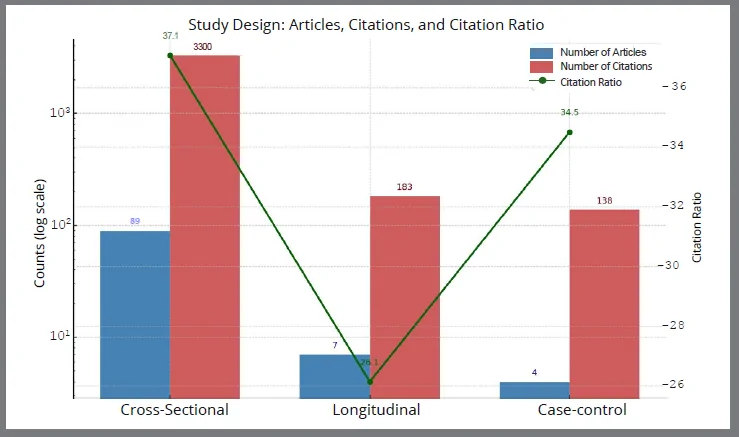
Distribution of studies by design. Blue bars indicate the number of publications per design, red bars the total number of citations, and the green line represents the citation-to-publication ratio (citations per paper). A logarithmic scale was applied to the Y-axis

**Figure 6:**
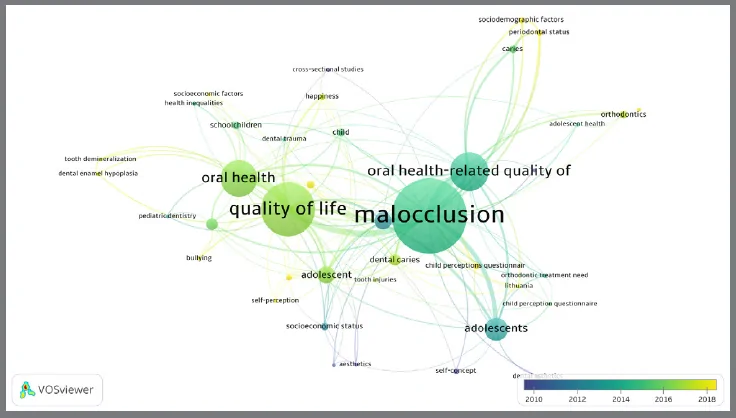
Keyword co-occurrence over time. Visualization of keywords appearing at least twice among the top 100 most-cited articles.

Among the 100 most-cited articles, cross-sectional studies predominated (n = 89), accounting for 3,300 citations (Fig. 5). Author-defined keywords with more than two occurrences are presented in Figure 6. Of 121 keywords identified, 40 met the threshold. The most frequent were “malocclusion” (n = 39), “quality of life” (n = 28), “oral health-related quality of life” (n = 20), “oral health” (n = 19), and “adolescents” (n = 12). As a Keywords Plus term, “malocclusion” had the highest citation count among the top 10 most-cited articles across universities (8%), followed by “children” (7%) and “orthodontic treatment need” (6%) (Fig. 3B).

Table 2 summarizes the content analysis of the 100 most-cited articles on the biopsychosocial impact of malocclusion in school-aged children and adolescents. Brazilian school-aged youth were the predominant sample (54 publications), with the 11-14-year age range most frequently studied (17 studies). The Dental Aesthetic Index (DAI) was the most frequently used tool for malocclusion assessment, followed by other recognized indices such as the Index of Orthodontic Treatment Need (IOTN). Psychological outcomes were the most frequently reported and included oral health-related quality of life (OHRQoL), self-esteem, self-image, self-concept, emotional well-being, and personal satisfaction. OHRQoL was predominantly assessed using the Child Perceptions Questionnaire (CPQ), with other validated instruments, including the Oral Aesthetic Subjective Impact Scale (OASIS) (Table 2).

**Table 2:** Summary of the content analysis of the 100 most-cited papers on the biopsychosocial impact of malocclusion in school-aged children and adolescents.

Rank	Study sample	Age group	Sample size	Malocclusion diagnosis indices	Biopsychosocial outcomes	Assessment instruments	Impact of malocclusion
1	Schoolchildren from Australia^19^	8-13	677	DAI	OHRQoL	CPQ_8-10_ and CPQ_11-14_	Children aged 8-10 years with malocclusion reported significantly poorer OHRQoL (B = 4.78, p < 0.001).
2	Schoolchildren from The United Kingdom^20^	14-15	334	IOTN-AC and IOTN-DHC	Self-perception of dental esthetics	OASIS	Children with poorer anterior tooth aesthetics, as assessed by the examiner, were significantly more likely to report a negative self-perception (p < 0.001).
3	Schoolchildren from Brazil^21^	10-14	333	DAI	OHRQoL Self-perception of dental esthetics Self-esteem	OIDP OASIS GSE	Children with upper anterior crowding ≥2 mm or a pronounced need for orthodontic treatment had greater aesthetic impact from malocclusion (OR = 2.0 and OR = 4.3; p ≤ 0.028).
4	Schoolchildren from Brazil^22^	13-20	301	DAI	OHRQoL Psychosocial Impact of Dental Aesthetics Body Satisfaction	OHIP-14 PIDAQ BSS	Children with very severe malocclusion experienced greater psychosocial and oral health-related impacts, with higher dissatisfaction with appearance significantly linked to increased dental aesthetic concerns (PIDAQ mean = 24.9; OHIP-14 β = 0.405; BSS β = 0.401; p < 0.001).
5	Schoolchildren from Brazil^23^	12	515	DAI	OHRQoL	CPQ_11-14_	Children with an orthodontic treatment need had a significantly greater negative impact on oral health-related quality of life (PR = 1.12; p = 0.0019).
6	Schoolchildren from Canada^24^	11-14	370	IOTN-AC	OHRQoL	CPQ_11-14_	Higher AC-IOTN scores were linked to poorer oral health-related quality of life and increased CPQ_11-14_ scores (β = 0.128; OR = 1.22; p < 0.01).
7	Schoolchildren from India^25^	10-17	1.140	IOTN-AC and IOTN-DHC	Self-esteem	RSES	Stepwise regression showed that DHC, AC, dental decay, tooth loss in aesthetic/masticatory zones, and anterior tooth fracture predicted lower self-esteem (p < 0.05).
8	Schoolchildren from Jordania^26^	11-12	920	Self-perception	Bullying	Modified questionnaire by Shaw et al. (1980)	Of 433 bullying reports, the most targeted dental features were spaced/missing teeth (21.5%), tooth shape/color (20.6%), and prominent front teeth (19.6%).
9	Schoolchildren from Brazil^27^	8-10	1.204	DAI	OHRQoL	CPQ_8-10_	Malocclusion was significantly associated with poorer OHRQoL (PR = 1.30; 95% CI: 1.15-1.46; p < 0.001), particularly anterior spacing and increased maxillary overjet (p < 0.001).
10	Schoolchildren from Jordania^28^	14-16	400	IOTN-AC and IOTN-DHC	Perceived need, satisfaction with appearance, and social impact of malocclusion Self-esteem	Direct questions and questionnaire by Mandall et al. (1999) GSE	Perceived orthodontic need was linked to higher normative need (AC: β = 0.209, p < 0.001; DHC: β = 0.153, p = 0.003), greater dissatisfaction with dental appearance (β = 0.320, p < 0.001), and hiding the smile (β = 0.176, p < 0.001). Social impact correlated with perceived and normative need (ρ = 0.252-0.610, p < 0.05). Lower self-esteem was associated with higher perceived/normative need and poorer dental aesthetics (ρ = 0.134-0.204, p < 0.05). Dissatisfaction with appearance (p < 0.001), hiding the smile (p = 0.001), and belief that straight teeth increase popularity (p = 0.022) were the main predictors of low self-esteem.
11	Schoolchildren from Norway^29^	11	359	IOTN-AC and IOTN-DHC	Satisfaction with dental appearance and desire for treatment Self-esteem	Modified questionnaire by Fox et al. (1982) GSE	Children with malocclusion reported greater orthodontic concern (p < 0.001) and expressed a stronger desire for treatment than dissatisfaction with appearance (p < 0.001). Higher concern was also associated with lower self-esteem (p < 0.01).
12	Schoolchildren from Italia^30^	n.a.	516	Molar relationship, posterior crossbite or scissor bite, overjet and overbite, crowding, and diastemas	Self-esteem.	MSCS	Children with dental crowding or posterior crossbite showed significantly lower MSCS scores across multiple domains (social, competence, physical, academic, global; all p ≤ 0.019) within the pathological range (≤ 86). Crowding increased odds of abnormal global self-concept (OR = 5.36; 95% CI: 3.49-8.23), and posterior crossbite further elevated these odds (OR = 6.15; 95% CI: 3.55-10.68).
13	Schoolchildren from Brazil^31^	11-14	594	Deep overbite, increased overjet, anterior open bite, anterior crossbite, and posterior crossbite	OHRQoL	CPQ_11-14_ISF:16	Children with malocclusion showed significantly poorer emotional (RR = 1.36; 95% CI: 1.14-1.61) and social well-being (RR = 1.40; 95% CI: 1.17-1.69), as well as greater overall negative impact on OHRQoL (RR = 1.18; 95% CI: 1.06-1.31).
14	Schoolchildren from Brazil^32^	11-14	509	DAI	OHRQoL	CPQ_11-14_ISF:16	Greater malocclusion severity was significantly associated with higher CPQ_11-14_ scores (β = 0.85; 95% CI: 0.22-1.48), reflecting increased negative impact on emotional and social well-being, with no significant effect on oral symptoms or functional limitation.
15	Schoolchildren from Brazil^33^	11-12	571	Criteria by Helm et al. (1985)	OHRQoL	Child-OIDP	Children perceiving dental position problems had 2.4 times higher odds of negative impact on oral health-related quality of life (Child-OIDP > 0; OR = 2.4).
16	Schoolchildren from Brazil^34^	8	728	Criteria by Grabowski et al. (2007)	OHRQoL	CPQ_8-10_	After adjusting for sociodemographic and clinical factors, malocclusion was not significantly associated with any OHRQoL domain.
17	Schoolchildren from Brazil^35^	11-14	509	DAI	OHRQoL	CPQ_11-14_ISF:16	After adjusting for socioeconomic and clinical factors, malocclusion was not significantly associated with OHRQoL.
18	Schoolchildren from Brazil^36^	8-10	750	DAI	OHRQoL	CPQ_8-10_	Children with very severe malocclusion had higher CPQ_8-10_ scores (p = 0.033), but malocclusion was not significant in the adjusted regression, showing an effect only in unadjusted analysis.
19	Schoolchildren from Thailand^37^	10-14	455	IOTN-AC	Sense of coherence Dental Coping Beliefs OHRQoL	SOC-13 DCBS CPQ_11-14_	Malocclusion was not significantly associated with OHRQoL at any follow-up (3, 6, or 9 months), despite good model fit (CMIN/DF = 2.601; SRMR = 0.056; CFI = 0.974; RMSEA = 0.059).
20	Schoolchildren from Spain^38^	12-15	627	IOTN-AC and IOTN-DHC	Psychosocial Impact of Dental Aesthetics	PIDAQ	Adolescents with severe malocclusion reported significantly worse dental aesthetics-related quality of life, with higher PIDAQ total and subscale scores and lower dental self-confidence (p < 0.05). IOTN-DHC independently predicted PIDAQ outcomes, with overjet, submerged teeth, severe displacement, and increased overbite showing the strongest associations.
21	Schoolchildren from Brazil^39^	8-10	1.201	DAI	OHRQoL	CPQ_8-10_	Anterior maxillary overjet > 4 mm was significantly linked to worse OHRQoL, both overall (bivariate OR = 1.75, p = 0.010; multilevel OR = 1.64, p = 0.037) and in specific domains: Functional Limitations (OR = 1.92, p = 0.005), Emotional Well-being (OR = 1.73, p = 0.027), and Social Well-being (OR = 1.66, p = 0.049). Other malocclusions (open bite, diastema, crowding) showed no significant effects.
22	Schoolchildren from Jordan^40^	11-12	920	Self-perception	OHRQoL Bullying	CPQ_11-14_ Modified questionnaire by Shaw et al. (1980)	Children bullied for their dental appearance had significantly higher CPQ_11-14_ scores than those not bullied, reflecting greater negative OHRQoL impact: total, oral symptoms, functional limitations, emotional well-being, and social well-being, all p < 0.001.
23	Schoolchildren from The United Kingdom^41^	Baseline: 11-12 Follow-up: 14-15	Baseline: 374/296 Follow-up: 217/173	IOTN-AC and IOTN-DHC	OHRQoL Self-esteem	CPQ_11-14_ISF:16 CHQ-CF87	Cross-sectional analysis showed that poorer self-rated dental aesthetics were linked to worse OHRQoL (β = 0.2; p < 0.001).
24	Schoolchildren from Brazil^42^	12	248	IOTN-AC and IOTN-DHC	OHRQoL Self-esteem Self-perception of dental esthetics	CPQ_11-14_ GSE OASIS	Self-perceived dental aesthetics closely matched normative assessments (p < 0.0001). Multilevel regression showed that poorer self-perception and lower self-esteem were strong predictors of higher IOTN-AC scores (p < 0.0001 and p = 0.028, respectively), independent of sex and OHRQoL. Normative treatment need also remained significant (p < 0.0001), indicating that children with negative self-perception and low self-esteem were more likely to truly require orthodontic treatment.
25	Schoolchildren from Brazil^43^	11-14	Case group: 405 Control group: 810	DAI	OHRQoL	CPQ_11-14_ISF:16	Adolescents with malocclusion had 1.68 times higher odds of reporting a high negative impact on OHRQoL than peers with none or mild malocclusion (adjusted OR = 1.68; 95% CI: 1.30-2.18; p < 0.001), independent of age, caries, and other confounders.
26	Schoolchildren from Brazil^44^	12-13	704	DAI	Dental Appearance Dissatisfaction Oral Function Dissatisfaction	Structured questionnaire	Adolescents with higher DAI scores had a 5% increased likelihood of dissatisfaction with dental appearance per point (OR = 1.05; 95% CI: 1.01-1.08; p = 0.001). Dissatisfaction was particularly linked to missing teeth (OR = 3.94), maxillary anterior irregularity ≥ 3 mm (OR = 3.48), and mandibular anterior irregularity ≥ 2 mm (OR = 2.88), while DAI scores were not associated with dissatisfaction regarding oral functions.
27	Schoolchildren from Brazil^45^	15-16	1.060	IOTN-DHC	OHRQoL	OIDP	Adolescents with definite orthodontic treatment needs had a higher prevalence of daily life impacts (42.5%) than those with moderate (20.7%) or no/slight needs (19.1%) (p < 0.001), mainly affecting smiling (15.8%) and speaking (9.2%). Among those impacted, 52.0% rated the effects as severe or very severe, especially in studying (100%) and emotional well-being (64.8%). Both impact extent (p = 0.005) and severity (p = 0.011) increased with greater treatment need, independent of socioeconomic status.
28	Schoolchildren from The United Kingdom^46^	11-12	439	IOTN-AC and IOTN-DHC	Self-perception of dental esthetics. Self-esteem.	OASIS Piers Harris questionnaire	Higher examiner-rated IOTN AC (ρ = 0.24; p < 0.001) and DHC grades (ρ = 0.18; p < 0.001) were linked to greater psychosocial impact. Self-esteem was negatively correlated with self-rated IOTN-AC (ρ = -0.14; p < 0.05) and OASIS (ρ = -0.35; p < 0.001), indicating that higher self-esteem corresponds to more positive dental self-perception and lower psychosocial impact. Children rated their dental appearance more favorably than examiners (p < 0.001).
29	Schoolchildren from New Zealand^47^	12-13	353	DAI	OHRQoL Mental Health / Self-esteem Propensity to Somatization Body Satisfaction	CPQ_11-14_ISF:16 Child Health CHQ-CF87 - Subdomain of Mental Health and Self-esteem Modified SCL-90 BIDQ	Higher DAI scores were significantly associated with poorer OHRQoL (β = 0.092; p < 0.01), with more severe malocclusion linked to greater daily life impacts, independent of caries. A structural equation model explained 62% of the variance in OHRQoL.
30	Schoolchildren from Brazil^48^	11-14	403	DAI	OHRQoL	CPQ_11-14_ISF:16	Malocclusion was not significantly associated with frequent negative impacts on OHRQoL after adjustment (PR = 1.33; 95% CI: 0.94-1.88; p = 0.107), despite higher crude prevalence among affected adolescents.
31	Schoolchildren from The United States^49^	n.a.	860	PAR	Children's attitudes and perceptions towards their occlusion and need for braces	Structured questionnaire	Clinical parameters such as overjet, anterior crowding, molar relationships, and soft tissue profile were significantly associated with the perceived need for orthodontic appliances.
32	Schoolchildren from Brazil^50^	Baseline: 12 Follow-up: 14	Baseline: 1.134 Follow-up: 747	DAI	OHRQoL	CPQ_11-14_	Adolescents with malocclusion had consistently higher CPQ11-14 scores over 2 years, indicating persistently poorer oral health-related quality of life, independent of sociodemographic and oral health factors.
33	Schoolchildren from Brazil^51^	11-14	509	DAI	Dental caries	DMFT	Adolescents with handicapping malocclusion had a 31% higher prevalence of dental caries (adjusted PR = 1.31; p = 0.032), with greater occurrence and severity in those with maxillary irregularity (p = 0.043; p = 0.021) and abnormal molar relationships (p = 0.024; p = 0.021).
34	Schoolchildren from New Zealand^52^	12-13	353	DAI	OHRQoL Mental Health / Self-esteem Propensity to Somatization Body Satisfaction	CPQ_11-14_ISF:16 Child Health CHQ-CF87 - Subdomain of Mental Health and Self-esteem Modified SCL-90 BIDQ	Adolescents with handicapping malocclusion had poorer OHRQoL and greater psychosocial impact, including higher somatization (p = 0.006) and appearance-related stress (p = 0.003), along with lower psychological well-being ( p < 0.001). Malocclusion severity was positively associated with negative oral health impact (DAI β = 0.10; p = 0.048), independent of sociodemographic and clinical factors (adjusted R² = 0.50).
35	Schoolchildren from Brazil^53^	15-16	Case group: 279 Control group: 558	IOTN-DHC	OHRQoL	OIDP	Adolescents with untreated malocclusion who had a definite treatment need were more likely to report condition-specific impacts (OR = 1.59; 95% CI: 1.01-2.50).
36	Schoolchildren from Brazil^54^	12-13	704	DAI	Dental Appearance Dissatisfaction Oral Function Dissatisfaction Desire for Orthodontic Treatment	Structured questionnaire	The likelihood of wanting orthodontic treatment increased with severity: elective need PR = 1.25 (95% CI: 1.12-1.39), severe PR = 1.38 (95% CI: 1.23-1.55), and very severe PR = 1.49 (95% CI: 1.32-1.69). Specific traits, 1 mm diastema PR = 1.24, ≥2 mm diastema PR = 1.34, maxillary irregularity ≥3 mm PR = 1.48, and overjet ≥6 mm PR = 1.29, were also significantly associated.
37	Schoolchildren from China^55^	15	364	IOTN-AC and IOTN-DHC DAI ICON PAR	OHRQoL	CPQ_11-14_	Adolescents with malocclusion (PAR) had higher total CPQ_11-14_ scores (p = 0.031), particularly in Functional Limitations and Social Well-being (p < 0.05). Very severe malocclusion (DAI) was associated with higher total CPQ scores (adjusted OR = 2.10; 95% CI: 1.06-4.13; p = 0.032), mainly affecting Functional Limitations.
38	Schoolchildren from Brazil^56^	7-10	1.256	Criteria by Foster & Hamilton (1969) and Grabowski et al. (2007)	OHRQoL	CPQ_8-10_	Bivariate analysis showed poorer OHRQoL was associated with increased overjet (OR = 1.62; 95% CI: 1.23-2.14; p = 0.0005), with borderline relevance for deepbite and Class II malocclusion. Multilevel analysis confirmed increased overjet (adjusted OR = 1.60; 95% CI: 1.36-1.90; p < 0.0001) as significant determinants of poorer OHRQoL.
39	Schoolchildren from The United States^57^	8-11	1.566	IOTN-AC and IOTN-DHC	OHRQoL	Modified version of the Michigan Oral HealthRelated Quality of Life Scale - Child Version	Correlation analyses revealed significant associations between children’s self-perceptions and provider assessments (p ≤ 0.008). Older children reported lower satisfaction with dental appearance (OHRQoL psychological domain; r = −0.055; p = 0.028) and a greater desire for orthodontic treatment (r = 0.080; p = 0.037).
40	Schoolchildren from Brazil^58^	8-14	167	DAI	OHRQoL Psychological Well-being - Anxiety and Depression	CPQ_8-10_ and CPQ_11-14_ RCMAS CDI	Bivariate analysis found no significant association between malocclusion and total CPQ scores in children aged 8-10 or 11-14 years.
41	Schoolchildren from Mexico^59^	8-10	212	DAI	OHRQoL	CPQ_8-10_	Bivariate analysis showed no significant differences in CPQ_8-10_ scores among malocclusion groups (p > 0.05). After adjusting for age and sex, severe malocclusion was significantly associated with poorer OHRQoL, with affected children 5.2 times more likely to have higher CPQ_8-10_ scores (OR = 5.2; 95% CI: 1.13-24.1; p = 0.034).
42	Schoolchildren from Brazil^60^	13-20	301	DAI	Psychosocial Impact of Dental Aesthetics	PIDAQ	Mean PIDAQ scores increased with DAI grades (p < 0.001). Multiple regression showed DAI was positively associated with total PIDAQ (β = 0.158; p = 0.003) and with dental self-confidence (β = 0.174; p < 0.001) and psychological impact (β = 0.146; p = 0.006) subscales. Dissatisfaction with dental appearance was the strongest predictor of negative dental aesthetic quality of life (β = 0.531; p < 0.001).
43	Schoolchildren from Brazil^5^	11-14	1.612	DAI	OHRQoL	CPQ_11-14_ISF:16	Malocclusion was significantly linked to greater negative impact on the Emotional and Social Well-Being domains of CPQ_11-14_, with adolescents showing progressively higher risk as severity increased (PR = 1.28-1.61; p < 0.05). Overall CPQ scores were also worse for definite (PR = 1.11; p = 0.022) and handicapping malocclusion (PR = 1.26; p < 0.001) after adjusting for sociodemographic and clinical factors.
44	Schoolchildren from Brazil^61^	8-10	Case group: 182 Control group: 364	DAI	OHRQoL	CPQ_8-10_	There was no significant difference in malocclusion prevalence between cases and controls (p = 0.230), and adjusted analysis also showed no significant association with high negative impact on OHRQoL (p = 0.327).
45	Schoolchildren from Brazil^62^	14-18	403	DAI	OHRQoL Self-perception of dental esthetics Self-esteem	OIDP OASIS GSE	Malocclusion was significantly linked to negative aesthetic impact, especially with upper anterior crowding >2 mm (p < 0.001), median diastema >2 mm (p = 0.019), missing anterior teeth (p = 0.041), and upper anterior overjet >4 mm (p < 0.001). Key risk factors identified in logistic regression were upper anterior crowding >2 mm (adjusted OR = 2.01; p = 0.009), median diastema >2 mm (adjusted OR = 2.26; p = 0.040), and high normative treatment need (adjusted OR = 2.90; p < 0.001).
46	Schoolchildren from China^63^	12	589	IOTN-AC and IOTN-DHC DAI ICON PAR	OHRQoL	CPQ_11-14_ISF:8 and CPQ_11-14_RSF:8	Adjusted ordinal regression showed that severe and very severe malocclusion (DAI) were associated with higher total CPQ_11-14_ scores (aOR = 1.59 and 1.90, respectively).
47	Schoolchildren from Brazil^64^	12	1.134	DAI	OHRQoL Subjective Happiness	CPQ_11-14_ISF:16 SHS	Severe and handicapping malocclusion increased the risk of poorer overall quality of life (RR = 1.20 and 1.26) and negatively impacted Emotional (RR = 1.44 and 1.47) and Social Well-Being (RR = 1.43 and 1.57). Children with definite malocclusion also reported lower subjective happiness (RR = 0.97).
48	Schoolchildren from Peru^65^	11-14	473	Angle’s classification	OHRQoL	CPQ_11-14_	Class III malocclusion was associated with lower negative impact on Emotional Well-Being (RR = 0.71; p < 0.01). Class II malocclusion showed no significant association with total or domain-specific CPQ_11-14_ scores.
49	Schoolchildren from Brazil^66^	8-14	145	DAI	OHRQoL Psychological Well-being - Anxiety and Depression	CPQ_8-10_ and CPQ_11-14_ RCMAS CDI	Children with handicapping severe malocclusion showed higher anxiety (RCMAS = 16.92) and lower diurnal cortisol decline (0.13 µg/dl), indicating greater physiological stress. Although those with definitive malocclusion reported poorer oral health and slightly lower overall quality of life, differences were not statistically significant, and DAI was not a significant predictor in the overall well-being model (β = 0.138; p = 0.087).
50	Schoolchildren from Brazil^67^	8-10	102	DAI	OHRQoL	CPQ_8-10_	Malocclusion was linked to toothache, eating and speaking difficulties, emotional distress, and social challenges. Children with anterior irregularities or crossbites reported being bothered by their appearance, feeling sad, struggling in class, and having trouble reading aloud or participating in games. Overall, worse malocclusion correlated with higher CPQ_8-10_ scores (p = 0.034).
51	Schoolchildren from Brazil^68^	14-18	300	DAI	Dental Appearance Dissatisfaction Oral Function Dissatisfaction Perception of the Need for Orthodontic Treatment	Structured questionnaire	Incisal crowding (OR = 2.8; p < 0.01) and overjet ≥4 mm (OR = 2.4; p < 0.01) were strongly linked to dissatisfaction with dental appearance. Perceived need for orthodontic treatment was significantly associated with mandibular anterior irregularity ≥2 mm (OR = 3.3; p < 0.01), overjet ≥4 mm (OR = 1.7; p = 0.05), and diastema ≥2 mm (OR = 3.1; p = 0.01).
52	Schoolchildren from Nigeria^69^	11-14	248	DAI	OHRQoL	CPQ_11-14_	CPQ showed no significant correlation with DAI (r = −0.019; p = 0.760), indicating malocclusion severity was not directly linked to self-reported OHRQoL.
53	Schoolchildren from Brazil^70^	8-10	390	DAI	OHRQoL	CPQ_8-10_	Overall, malocclusion was not significantly associated with OHRQoL (p = 0.212). However, in the multivariate analysis, anterior crossbite (PR = 1.28; p < 0.001) and mandibular crowding (PR = 1.36; p = 0.025) remained significant predictors of negative OHRQoL impact.
54	Schoolchildren from Brazil^71^	9-10	1.204	DAI	OHRQoL	CPQ_8-10_	Children with malocclusion had higher CPQ_8-10_ scores (p = 0.001) and worse emotional (p < 0.001) and social well-being (p = 0.004). Adjusted analysis showed malocclusion increased negative OHRQoL by 1.33 times (PR = 1.33; 95% CI: 1.12-1.59; p = 0.001)
55	Schoolchildren from Brazil^72^	12	1.134	DAI	OHRQoL Subjective Happiness	CPQ_11-14_ISF:16 SHS	Adjusted analysis showed that children with malocclusion had lower subjective happiness (adjusted ratio = 0.98; 95% CI: 0.96-0.99). Poorer OHRQoL was negatively correlated with happiness (p < 0.001), indicating malocclusion is linked to reduced well-being.
56	Schoolchildren from Nigeria^73^	12-18	520	DAI	Self-esteem	GSE	Malocclusion severity was significantly correlated with orthodontic concern (p < 0.01).
57	Schoolchildren from Brazil^74^	12	1.134	DAI	OHRQoL	CPQ_11-14_ISF:16	Children with malocclusion had higher CPQ_11-14_ scores. Malocclusion was associated with a 12% greater impact in adjustment analysis (RR = 1.12; 95% CI: 1.07-1.17).
58	Schoolchildren from Japan^75^	10-16	420	IOTN-DHC	OHRQoL	CPQ_11-14_	Increased overjet was linked to oral symptoms (β = 0.66), functional limitations (β = 0.62), and social well-being (β = 0.50), while deep bite was associated with oral symptoms (β = 0.54) and functional limitations (β = 0.45). Anterior crossbite, posterior crossbite, and crowding, showed no significant effects.
59	Schoolchildren from Zambia^76^	12-14	384	DAI	OHRQoL	COHIP-SF19	Malocclusion significantly affected OHRQoL (p < 0.001). Anteriomaxillary overjet had the greatest impact (p < 0.001). Anteroposterior molar relationship, anteriomandibular overjet, spacing, crowding, and diastema, also worsened OHRQoL (p ≤ 0.027), while missing maxillary teeth and vertical anterior open bite had no effect.
60	Schoolchildren from Brazil^77^	15-16	1.318	Maxillary overjet, Mandibular overjet, Openbite, Overbite, Centreline, Dental crowding, Dental spacing, Maxillary irregularity, Mandibular irregularity	OHRQoL	OIDP	In the multivariate model, independent predictors of condition-specific dental impact included maxillary overjet (aOR = 7.39/mm), mandibular overjet (aOR = 1.99), open bite (aOR = 1.39), midline deviation (aOR up to 1.98), spacing in one segment (aOR = 1.72), and maxillary irregularity (aOR = 1.17).
61	Schoolchildren from Egypt^78^	11-14	11.700	Overbite, Overjet, Anterior open bite, Crowding, Spacing, Anterior crossbite.	OHRQoL	CPQ_11-14_	Malocclusion severity was significantly linked to higher CPQ_11-14_ scores, with greater severity corresponding to progressively higher total scores.
62	Schoolchildren from Brazil^79^	8-10	Case group: 78 Control group: 312	DAI	Bullying.	Specific validated question from the CPQ_8-10_	Children with very severe malocclusion (aOR = 2.29; 95% CI: 1.03-5.10; p = 0.042) and maxillary misalignment ≥ 3 mm (adjusted OR = 2.23; 95% CI: 1.05-4.73; p = 0.038) were at higher risk of verbal bullying, showing a significant link between malocclusion severity and bullying.
63	Schoolchildren from Malaysia^80^	13-16	252	IOTN-DHC	OHRQoL	OHIP-14	A weak positive correlation was observed between malocclusion and OHRQoL (r = 0.186; p < 0.01), and regression showed that each increase in severity raised OHIP-14 scores 1.28-fold (p < 0.01), explaining 3.2% of the variance, indicating predominantly psychological effects.
64	Schoolchildren from Brazil^81^	12-15	1.015	Angle’s classification DAI Posterior crossbite, Posterior open bite and Deep overbite Self-perception	OHRQoL	OHIP-14	Normative orthodontic need increased negative impact on OHRQoL by 27% (PR = 1.27; 95% CI: 1.03-1.56), while self-perceived need increased it by 154% (PR = 2.54; 95% CI: 1.81-3.56), the strongest predictor. Other occlusal traits were not significant, indicating malocclusion severity strongly affects OHRQoL.
65	Schoolchildren from Brazil^82^	12-16	1.290	DAI	Dissatisfaction with smile	Standardized questionnaire	Dissatisfaction with the smile was significantly associated with missing anterior teeth, incisal spacing, diastema ≥ 2 mm, maxillary anterior irregularity ≥ 2 mm, mandibular anterior irregularity ≥ 2 mm, and vertical open bite ≥ 2 mm, all p ≤ 0.049. Crossbite was not significant. Adolescents with malocclusion had higher dissatisfaction prevalence (27.1%; PR = 1.52; p < 0.001).
66	Schoolchildren from Brazil^83^	12-15	717	DAI	Selfperceptions and perceived esthetic of malocclusion: speech capability, chewing capability and report of pain.	Semi-structured questionnaire	At 12, missing teeth and open bite increased speech problems (OR = 2.87), while spacing also impacted speech; pain was linked to missing teeth and mandibular irregularity. At 15, mandibular overjet strongly increased speech difficulty (OR = 4.02), molar relationship affected chewing (OR = 1.66), and pain was associated with overjet and sex.
67	Schoolchildren from India^84^	14-15	1.618	DAI	Perception of Aesthetics, Function, and Speech.	Structured questionnaire	Objective malocclusion severity showed weak but significant correlations with adolescents’ perceptions of aesthetics, function, speech, and treatment need, with the strongest for aesthetics and speech (r = 0.489) and weakest for chewing (r = 0.109).
68	Schoolchildren from Brazil^85^	Baseline: 12 Follow-up: 14	Baseline: 1.134 Follow-up: 770	DAI	OHRQoL Subjective Happiness	CPQ_11-14_ISF:16 SHS	Adolescents with severe malocclusion had slightly but significantly lower happiness scores than those with mild or normal malocclusion (coefficient = −0.08; p = 0.044), showing that greater malocclusion severity is linked to reduced subjective happiness.
69	Schoolchildren from Peru^86^	14-20	1.062	ICON	OHRQoL	COHIP-SF19	Greater malocclusion severity was significantly linked to poorer OHRQoL in children (β = −0.059; p < 0.001), especially affecting oral health, social-emotional well-being, and self-image.
70	Schoolchildren from Jordan^87^	12	1.652	IOTN-DHC	OHRQoL	CPQ_11-14_ISF:16	Malocclusion had the strongest negative impact on OHRQoL (aOR = 12.76; 95% CI: 7.18-22.68; p < 0.001).
71	Schoolchildren from India^88^	12-15	900	DAI	OHRQoL	OIDP	Malocclusion significantly affected children’s OHRQoL, with high odds ratios for eating (OR = 8.9), cleaning teeth (OR = 13.1), emotional well-being (OR = 8.1), smiling (OR = 17.1), studying (OR = 2.1), and social contact (OR = 14.0). Oral impacts were common (60%), particularly in eating, cleaning teeth, and smiling, with tooth position being a major contributing factor.
72	Schoolchildren from The United States^89^	8-11	1.566	IOTN-AC and IOTN-DHC Angle’s classification	OHRQoL	Modified version of the Michigan OHRQoL scale-child	Objective and subjective orthodontic treatment need showed weak but significant correlations with smile-related quality of life (r = -0.145 to -0.177; p < 0.001). Treatment recommendation and desire for braces were also weakly yet significantly correlated with these outcomes (r = 0.080 to -0.141; p ≤ 0.002).
73	Schoolchildren from Brazil^90^	8-10	787	IOTN-AC and IOTN-DHC	Self-perception of dental esthetics	OASIS	Midline diastema (OR = 1.34; 95% CI: 1.01-1.79; p = 0.043), decreased anterior overbite (OR = 1.46; 95% CI: 1.03-2.07; p = 0.031), and increased anterior overbite (OR = 1.54; 95% CI: 1.09-2.18; p = 0.013) were significantly linked to greater aesthetic concern. After adjustment, increased overbite raised the odds of aesthetic impact by 1.54 (95% CI: 1.08-2.17; p = 0.018) and decreased overbite by 1.38 (95% CI: 0.97-1.96; p < 0.05).
74	Schoolchildren from Brazil^91^	11-14	332	IOTN-AC and IOTN-DHC	OHRQoL Self-esteem Self-perception of dental esthetics	OHIP-14 GSE OASIS	There was no statistically significant association between severe malocclusion and negative impact on OHRQoL (adjusted OR = 1.39; p = 0.20). Facial profile also had no effect (p = 0.92), whereas greater aesthetic concern significantly increased the likelihood of poorer quality of life 3.43-fold (p < 0.001).
75	Schoolchildren from Brazil^92^	12	406	DAI	OHRQoL Self-esteem	CPQ_11-14_ISF:16 RSES	Multiple regression showed that DAI alone was not significantly associated with OHRQoL (p = 0.905). However, self-esteem significantly modified this relationship (p = 0.023): in children with mild to moderate malocclusion, lower self-esteem was linked to greater OHRQoL impact, whereas this effect was not seen in severe cases. Self-esteem did not mediate the DAI-OHRQoL relationship (p = 0.662).
76	Schoolchildren from Spain^93^	12-16	1.158	IOTN-AC and IOTN-DHC DAI	Psychosocial Impact of Dental Aesthetics	PIDAQ	Regression analysis showed that malocclusion severity, measured by DAI, IOTN-DHC, and IOTN-AC, significantly predicted total PIDAQ scores and all domains (p < 0.01). Mean PIDAQ scores increased with severity, indicating that greater malocclusion worsens adolescents’ aesthetic and psychosocial self-perception.
77	Schoolchildren from China^94^	Baseline: 12 Follow-up 1: 15 Follow-up 2: 18	Baseline: 589 Follow-up 1: 364 Follow-up 2: 300	IOTN-AC and IOTN-DHC DAI ICON PAR	OHRQoL	CPQ_11-14_ISF:8 OHIP-14	Longitudinal analysis showed that only severe and very severe malocclusions significantly worsened OHRQoL, with adjusted relative risks of 1.09 (p = 0.032) and 1.17 (p = 0.001), respectively. Definitive orthodontic treatment need per IOTN-DHC was also linked to poorer OHRQoL (adjusted RR = 1.09, p = 0.019).
78	Schoolchildren from Lithuania^95^	11-18	911	IOTN-DHC	OHRQoL	CPQ_11-14_	Negative binomial regression showed that each one-point increase in IOTN worsened CPQ scores: total score increased by 8.6%, emotional domain by 15.8%, and social domain by 20.5% (p < 0.05)
79	Schoolchildren from India^96^	10-14	604	IOTN-AC and IOTN-DHC	OHRQoL	CPQ_11-14_	Correlations showed that malocclusion mainly affected girls’ emotional and social CPQ domains (p < 0.05). Perceived dental alignment was significantly associated with all CPQ domains (p < 0.001), and lack of orthodontic treatment strongly correlated with oral symptoms, functional limitations, and emotional and social well-being (p ≤ 0.03).
80	Schoolchildren from Lithuania^97^	11-18	1.510	Self-perception	Global Life Satisfaction OHRQoL	GLS CPQ	After adjusting for gender, age, and socioeconomic status, the association between self-perceived malocclusion and low global life satisfaction was not statistically significant (OR = 1.13; p = 0.147).
81	Schoolchildren from India^98^	11-14	1.130	Angle’s classification, oligodontia or gross orthodontic problems.	OHRQoL	CPQ_11-14_	Malocclusion significantly affected OHRQoL, with a direct effect of β = 0.076 (95% CI: 0.023-0.140; p = 0.011), indicating a higher perceived impact on quality of life.
82	Schoolchildren from Brazil^99^	12	201	IOTN-AC and IOTN-DHC DAI	OHRQoL	OIDP	Malocclusion had a greater impact on daily activities in children with higher normative orthodontic treatment needs (IOTN, p = 0.045; DAI, p = 0.009), although it was not associated with the total Child-OIDP score.
83	Schoolchildren from Brazil^100^	12-15	494	IOTN-AC DAI	Bullying OHRQoL	PeNSE CPQ_11-14_	Malocclusion was not directly linked to bullying history, but increased maxillary overjet negatively affected adolescents’ self-perception, indicating a potential risk factor for bullying.
84	Schoolchildren from Brazil^101^	12-15	1.172	IOTN-AC and IOTN-DHC DAI	Self-perception of dental esthetics	OASIS	Adolescents with orthodontic treatment need per DAI were more likely to perceive aesthetic impact (IOTN-AC: OR = 2.62, 95% CI: 1.77-3.89, p < 0.0001; OASIS: OR = 1.52, 95% CI: 1.21-1.93, p = 0.0004), while IOTN-DHC showed no significant association.
85	Schoolchildren from Iran^102^	12-15	900	DAI	Self-perceived treatment need and satisfaction with dental appearance	Adapted questionnaire by Shue-Te Yeh et al. (2000) and Kerosuo et al. (2004).	Children with malocclusion reported greater dissatisfaction with dental appearance, more difficulty chewing and speaking, and higher anxiety when smiling or teased.
86	Schoolchildren from Brazil^103^	10-15	389	DAI	OHRQoL	OIDP	Among daily life impacts, only overjet >3 mm was significantly associated with difficulty in teeth cleaning (PR 1.62; 95% CI 1.04-2.53; p = 0.03); other malocclusion traits showed no significant effects on OIDP dimensions.
87	Schoolchildren from Brazil^104^	12-15	615	DAI	Sense of coherence Self-perception of dental esthetics	SOC OASIS	Adolescents with malocclusion and negative self-perceived dental aesthetics had lower sense of coherence than those without treatment need and positive self-perception, indicating an impact on aesthetic self-perception and psychological well-being (p = 0.033).
88	Schoolchildren from Brazil^105^	11-14	463	DAI	OHRQoL	CPQ_11-14_ISF:16	Malocclusion was linked to a greater negative impact on children’s OHRQoL, especially in functional limitation, emotional well-being, and overall CPQ scores ( p ≤ 0.010).
89	Schoolchildren from Mexico^106^	8-10	480	DAI	OHRQoL	CPQ_8-10_	Malocclusion severity was linked to OHRQoL, with severe/very severe cases showing a 3.09-fold higher CPQ_8-10_ score and consistent impacts across all domains (p < 0.001).
90	Schoolchildren from Brazil^107^	8-10	787	DAI	OHRQoL	CPQ_8-10_	Anterior malocclusion traits, including crowding, spacing, midline diastema, overjet, and open bite, were not significantly associated with negative impacts on OHRQoL, even after multivariate adjustment (all p > 0.05).
91	Schoolchildren from Lithuania^108^	15-18	600	ICON	OHRQoL	CPQ	Adolescents with malocclusion showed poorer OHRQoL, with significant impacts on the emotional (p < 0.001) and social p < 0.001) domains of the CPQ, while oral symptoms (p = 0.292) and functional limitations (p = 0.316) were not affected.
92	Schoolchildren from Brazil^109^	11-14	775	DAI	OHRQoL	CPQ_11-14_ISF:16	CPQ_11-14_ scores increased with malocclusion severity, showing a trend toward poorer OHRQoL, particularly in the emotional and social domains, without reaching statistical significance after adjustment (p = 0.097).
93	Schoolchildren from Malaysia^110^	12-17	901	IOTN-AC	Psychosocial Impact of Dental Aesthetics	PIDAQ	Adolescents with self-perceived malocclusion showed greater psychosocial impacts related to dental aesthetics, including lack of dental self-confidence, social impact, psychological impact, and aesthetic concern.
94	Schoolchildren from Lithuania^111^	11-15	5.632	Self-perception	Orthodontic complaints	Structured Questionnaire	Multivariate analysis indicated that gender was the only factor significantly associated with self-reported malocclusion, with girls being 1.59 times more likely than boys to report orthodontic problems. No other demographic or socioeconomic factors showed a significant association with malocclusion complaints.
95	Schoolchildren from Nigeria^112^	12-17	274	ICON	OHRQoL	OHIP-14	Orthodontic treatment complexity was not significantly associated with OHRQoL (P = 0.106), but the presence of treatment need was linked to a higher impact on quality of life (P = 0.020). Among daily impacts, physical pain was the most frequent (34.7%), followed by psychological discomfort and physical limitations, while handicap dimensions were less affected.
96	Schoolchildren from Brazil^113^	Baseline: 12 Follow-up 1: 14 Follow-up 2: 18	Baseline: 1.134 Follow-up 1: 770 Follow-up 2: 768	DAI	OHRQoL	CPQ_11-14_ISF:16	Malocclusion was significantly associated with poorer OHRQoL over time (IRR = 1.15; 95% CI: 1.12-1.18).
97	Schoolchildren from Nigeria^114^	11-14	200	IOTN-AC	OHRQoL Malocclusion Impact	CPQ_11-14_ISF:16 MIQ	Adolescents with malocclusion had poorer OHRQoL scores, with a significant association in the adjusted regression (IRR = 1.15; 95% CI: 1.12-1.18), indicating a 15% higher likelihood of negative impact, despite the low correlation between subjective and objective assessments (p < 0.001).
98	Schoolchildren from Colombia^115^	13-16	387	DAI	Self-esteem	RSES	There was a weak but statistically significant negative correlation between malocclusion severity and self-esteem (p < 0.05), observed only among girls (p = 0.02).
99	Schoolchildren from Brazil^17^	Baseline: 12 Follow-up: 14	Baseline: 415 Follow-up: 355	DAI	Self-esteem Sense of coherence Oral health beliefs	RSES SOC-13 Questionnaire proposed by Broadbent et al. (2006)	Greater overjet was associated with poorer psychosocial status (β = −0.203; p < 0.05).
100	Schoolchildren from Brazil^18^	8-10	785	DAI IOTN-AC	Self-perception of dental esthetics	OASIS	Diastema significantly influenced aesthetic concern (p = 0.024), and the combination of diastema with crowding was also significant (p = 0.044), while crowding alone was not (p = 0.468), indicating that diastema increases perceived aesthetic impact.

n.a.: not applicable; aOR: adjusted odds ratio; B: unstandardized regression coefficient; BIDQ: Body Image Disturbance Questionnaire; BSS: Body Satisfaction Scale; CDI: Children’s Depression Inventory; CFI: Comparative Fit Index; CHQ-CF87: Child Health Questionnaire - Child Self-Report Form; CI: confidence interval; CMIN/DF: Chi-square Minimum/Degrees of Freedom; COHIP-SF19: Child Oral Health Impact Profile - Short Form 19; CPQ: Child Perception Questionnaire; DAI: Dental Aesthetic Index; DCBS: Dental Coping Beliefs Scale; DMFT: decayed, missing, and filled teeth; GLS: Child’s Global Life Satisfaction; GSE: Global Negative Self-Evaluation; IOTN-AC: Index of Orthodontic Treatment Need - Aesthetic Component; IOTN-DHC: Index of Orthodontic Treatment Need - Dental Health Component; IRR: Incidence Rate Ratio; ISF: Impact Short Form; MIQ: Malocclusion Impact Questionnaire; MSCS: Multidimensional Self-Concept Scale; OASIS: Oral Aesthetic Subjective Impact Scale; OHIP-14: Oral Health Impact Profile; OHRQoL: Oral Health-Related Quality of Life; OIDP: Oral Impact on Daily Performance; OR: odds ratio; p < 0.05: statistically significant; PAR: Peer Assessment Rating; PeNSE: National Survey of School Health; PIDAQ: Psychosocial Impact of Dental Aesthetics Questionnaire; PR: prevalence ratio; R²: coefficient of determination; RCMAS: Revised Children’s Manifest Anxiety Scale; RMSEA: Root Mean Square Error of Approximation; RR: relative risk; RSES: Rosenberg Self-Esteem Scale; RSF: Regression Short Form; SCL: Subscale of Somatization of the Symptom Checklist; SHS: Subjective Happiness Scale; SOC: Sense of Coherence Scale; SRMR: Standardized Root Mean Square Residual; β: standardized coefficient; ρ: Spearman’s correlation coefficient; RR = Rate Ratio.

Overall, reviewed studies indicated that malocclusion severity is associated with poorer OHRQoL, particularly in emotional and social domains. Traits such as increased overjet, diastemas, anterior crowding, and posterior crossbite were most frequently linked to higher aesthetic concern, bullying experiences, and greater demand for orthodontic treatment. Adolescents’ self-perception, expressed as dissatisfaction with dental appearance and lower self-esteem, was as influential as or even more than normative need in determining psychosocial impact (Table 2).

Poisson regression analysis (Table 3) revealed that sample size and publication year were significantly associated with citation counts. Studies with larger samples (>597) received fewer citations than smaller studies (adjusted RR = 0.73; 95% CI: 0.55-0.97; p = 0.033). Similarly, recent publications (2016-2024) were cited less than older ones (1996-2015; adjusted RR = 0.47; 95% CI: 0.34-0.64; p < 0.001). No significant associations were found for continent of origin or journal impact factor after adjustment.

**Table 3:** Poisson regression analysis between the independent variables and the number of article citations in Web of Science.

Variable	Crude		Adjusted	
	RR (95% CI)	p-value	RR (95% CI)	p-value
Continent				
South America	1		1	
Africa	0.50 (0.19; 1.33)	0.171	0.44 (0.19; 1.01)	0.055
Asia	0.94 (0.58; 1.52)	0.799	1.00 (0.66; 1.51)	0.995
Europe	1.06 (0.67; 1.66)	0.815	1.00 (0.68; 1.47)	0.991
North America	1.13 (0.58; 2.22)	0.714	0.91 (0.51; 1.62)	0.751
Oceania	1.69 (0.87; 3.32)	0.127	1.38 (0.78; 2.44)	0.277
Sample size				
< 597	1	0.046^*^	1	0.033^*^
> 597	0.71 (0.51; 0.99)		0.73 (0.55; 0.97)	
Year				
1996 - 2015	1	< 0.001^***^	1	< 0.001^***^
2016 - 2024	0.45 (0.33; 0.62)		0.47 (0.34; 0.64)	
Impact factor				
> 2.7	1	0.792	-	
≤ 2.7	1.05 (0.74; 1.48)			

CI: confidence interval; p-value: significance level; RR: rate ratio; ^*^p < 0.05; ^**^p < 0.01; ^*^p < 0.001.

## DISCUSSION

This study represents the first comprehensive bibliometric analysis of the 100 most-cited articles on the biopsychosocial impact of malocclusion in school-aged children and adolescents, mapping trends, key contributors, and thematic focuses. The included articles accumulated 3,621 citations, with cross-sectional studies predominating (n = 89), indicating a strong reliance on observational designs. Brazilian authors and institutions were prominently represented, with the Universidade Federal de Minas Gerais, Universidade Estadual de Campinas, and Universidade Federal de Santa Maria among the most productive institutions. Brazil led in both publication and citation counts, followed by the United Kingdom and Jordan, reflecting a concentration of research output in specific geographic regions. 

The number of citations per article ranged from 5 to 148, with a mean of 36.21. These figures differ from those reported in a bibliometric analysis of the 100 most-cited articles on OHRQoL, in which the top-cited study received 949 citations in the Web of Science Core Collection database.^15^ This discrepancy may be attributed to differences in study objectives and eligibility criteria. As in the present review, cross-sectional studies were also the most prevalent design in that analysis.^15^

A total of 36 journals were represented in this analysis, and no significant association was observed between journal impact factor and citation counts after adjustment. In this sample, open-access availability was more frequent among journals with lower impact factors (≤ 2.7), which may partially explain the lack of association between impact factor and citations and underscores the role of accessibility in enhancing research visibility. 

Additionally, the regression analysis indicated that larger sample size and more recent publication year were associated with lower citation counts. This pattern appears to be primarily explained by the historical timing of publication rather than by sample size itself. Citation accumulation in this field peaked between 2010 and 2014, particularly in 2013, a period marked by studies that established key conceptual and methodological frameworks for assessing the biopsychosocial impact of malocclusion. In contrast, most large-sample studies were published after 2015, placing them outside this period of highest citation density and limiting their opportunity for citation accrual. These findings suggest that citation impact is strongly shaped by publication timing and the developmental stage of the research field, rather than by methodological scale alone.

Citations, defined as references to prior studies acknowledging the origin of methods, ideas, and findings, are commonly used as an approximate indicator of scientific relevance.^116^ However, citation counts do not necessarily reflect the immediate impact of a publication, as they are strongly influenced by the interval between publication and recognition within the scientific community. Older articles tend to accumulate more citations due to longer exposure and progressive incorporation into subsequent research. In addition, the time required for the preparation, submission, and publication of citing articles directly affects citation accumulation. Previous studies indicate that citation frequency typically peaks within two to three years after publication, followed by a gradual decline, a phenomenon often described as “attention decay”.^117^ ^,^ ^118^ Accordingly, citation counts should be interpreted in light of exposure time and the dynamic process of scientific dissemination.

The journals with the highest number of publications and citations were *The Angle Orthodontist* and the *American Journal of Orthodontics and Dentofacial Orthopedics*, both specialized in orthodontics, with impact factors of 3.2 and 3.0, respectively, according to JCR® IF 2024. Although the impact factor is commonly used as an indicator to estimate a journal’s relative importance,^119^ analyses in this study showed that it did not have a statistically significant effect on the number of citations, indicating that articles published in journals with higher impact factors were not necessarily cited more frequently. Although Brazil leads in the number of published articles on the topic, none of the five studies published in the *International Journal of Environmental Research and Public Health*, which had the highest impact factor in this analysis (4.614), were authored by Brazilian researchers.

Of the 100 articles included in this bibliometric review, 89 used a cross-sectional design. This approach is favored for its lower cost, standardized and precise data collection, ability to estimate disease prevalence, control for confounding factors, and assess multiple outcomes within the same population.^120^ The school setting offers additional advantages by reducing selection bias associated with seeking dental care and providing access to a larger, more diverse sample of children and adolescents, enhancing representativeness and external validity. Nevertheless, cross-sectional designs are limited by the lack of temporal information, which restricts causal inference.^120^

The prominence of Brazilian authors and institutions in this bibliometric review highlights the strategic relevance of local research centers in promoting academic visibility. Universities such as the Federal University of Minas Gerais and the Federal University of Santa Maria act as central nodes in collaborative networks, supporting national leadership in the field. Scientific collaboration not only enhances productivity but also increases citation impact, as collaborative teams are more likely to produce influential work.^121^ ^-^ ^124^ The country’s prominent role in Latin American dental research also reflects a broader reconfiguration of the global geography of science, in which emerging economies are assuming an increasingly central role.^125^ ^,^ ^126^ This scenario reinforces the need for sustained investment in public research infrastructure and collaborative capacity to ensure long-term international scientific relevance.

At the same time, the concentration of publications in a limited number of countries may indicate a regional publication bias and draws attention to persistent global inequalities in scientific production. The underrepresentation of regions such as Africa and Oceania constrains the extrapolation of findings to diverse sociocultural contexts, where perceptions of dental aesthetics, social norms, access to orthodontic care, and school environments may differ substantially. Consequently, the generalizability of the biopsychosocial impacts of malocclusion identified in this review should be interpreted with caution, reinforcing the need for broader geographic representation and international collaboration in future research.

Content analysis demonstrated that malocclusion, predominantly assessed using the Dental Aesthetic Index (DAI), was consistently associated with poorer oral health-related quality of life (OHRQoL), particularly affecting the emotional, social, and physical domains. Studies showed that greater malocclusion severity measured by the DAI was associated with higher CPQ scores and worse emotional and social well-being among schoolchildren.^5^ ^,^ ^31^ ^,^ ^32^ Specific occlusal traits, including increased overjet, diastemas, and anterior crowding, were related to negative aesthetic self-perception and dissatisfaction with dental appearance.^39^ ^,^ ^62^ In addition, severe malocclusion was identified as a relevant factor associated with bullying experiences and increased demand for orthodontic treatment.^79^

A systematic review and meta-analysis investigating the impact of untreated malocclusion on OHRQoL showed that greater malocclusion severity is significantly associated with increased negative effects, especially in physical and psychosocial domains.^127^ Studies using the DAI, Index of Orthodontic Treatment Need (IOTN), or Index of Complexity, Outcome and Need (ICON) to assess malocclusion, together with the Oral Health Impact Profile (OHIP) to measure OHRQoL, indicated that more severe malocclusion corresponds to higher impact scores in physical limitations, pain, psychological discomfort, and social and psychological disability.^127^ Early prevention and treatment, including interceptive interventions, orthodontic follow-up, and oral health education, allow prioritization of high-impact cases, mitigate psychosocial consequences, and improve OHRQoL.

This study has some limitations. The exclusive use of the Web of Science Core Collection represents a methodological constraint, as relevant studies indexed in other databases, such as Scopus or Google Schoolar, may not have been captured. However, WoS-CC is widely recognized for its rigorous indexing criteria and standardized citation metrics, making it particularly suitable for bibliometric analyses focused on citation impact and network mapping. Additionally, the focus on the 100 most-cited articles may have excluded relevant emerging research that has not yet accumulated a high citation count.

Nevertheless, this analysis provides a comprehensive overview of influential trends in the field, offering valuable insights for researchers, oral health professionals, and policymakers seeking to integrate biopsychosocial perspectives into malocclusion prevention and intervention strategies. Future research should explore additional dimensions, including self-esteem, academic performance, and social relationships, incorporate underrepresented regions, and prioritize longitudinal and cohort designs to overcome the inherent limitations of cross-sectional studies and establish more robust causal relationships between malocclusion and biopsychosocial outcomes.

## CONCLUSIONS

This bibliometric analysis demonstrates that research on the biopsychosocial impact of malocclusion in school-aged children and adolescents is predominantly based on cross-sectional designs and emphasizes patient-reported outcomes, particularly oral health-related quality of life. The most influential studies consistently indicate that greater malocclusion severity is associated with adverse emotional, social, and psychological outcomes, including dissatisfaction with dental appearance, reduced self-esteem, and bullying experiences, underscoring the relevance of subjective perceptions alongside normative treatment need. The prominent contribution of Brazilian institutions highlights their central role in knowledge production, while the geographic concentration of highly cited studies indicates limited global representativeness. Overall, these findings reinforce the relevance of biopsychosocial frameworks in orthodontic research and practice and point to the need for longitudinal investigations to better inform preventive and therapeutic strategies aimed at reducing the psychosocial burden of malocclusion during childhood and adolescence.

## Data Availability

Due to the sensitive nature of the data and ethical restrictions, the datasets supporting this study are not publicly available.
